# miR-130b-3p Modulates Epithelial-Mesenchymal Crosstalk in Lung Fibrosis by Targeting IGF-1

**DOI:** 10.1371/journal.pone.0150418

**Published:** 2016-03-08

**Authors:** Shuhong Li, Jing Geng, Xuefeng Xu, Xiaoxi Huang, Dong Leng, Dingyuan Jiang, Jiurong Liang, Chen Wang, Dianhua Jiang, Huaping Dai

**Affiliations:** 1 Department of Respiratory and Critical Care Medicine, Beijing Key Laboratory of Respiratory and Pulmonary Circulation Disorders, Beijing Chao-Yang Hospital-Beijing Institute of Respiratory Medicine, Capital Medical University, Beijing 100020, P.R. China; 2 National Clinical Research Centre for Respiratory Medicine, Beijing Hospital, Beijing 100730, P.R. China; 3 Department of Medical Research, Beijing Chao-Yang Hospital, Capital Medical University, Beijing 100020, P.R. China; 4 Clinical Laboratory, Beijing Chao-Yang Hospital, Capital Medical University, Beijing 100020, P.R. China; 5 Department of Medicine Pulmonary Division and Women’s Guild Lung Institute, Cedars-Sinai Medical Center, Los Angeles, CA 90048, United States of America; 6 Department of Pulmonary and Critical Care Medicine, China–Japan Friendship Hospital, Beijing, 100029, P.R. China; University of Alabama at Birmingham, UNITED STATES

## Abstract

Idiopathic pulmonary fibrosis (IPF) is a chronic, progressive and usually lethal fibrotic lung disease with largely unknown etiology and pathogenesis. Evidence suggests microRNAs (miRNA) contribute to pathogenesis of IPF. In this study, we sought to identify miRNA expression signatures and determine the role of miR-130b-3p in lung fibrosis. The miRNA expression profile of the lungs from patients with IPF and normal donors was determined by Affymetrix microarray, and transcriptome with Affymetrix array. The functions and signal pathways as well as miRNA-mRNA networks were established by bioinformatics analysis. Luciferase assays and ELISA were used to confirm the miRNA target gene. The effect of miRNA-transfected epithelium on fibroblast activities was assessed using a co-culture system. The fibroblast activities were determined by qRT-PCR, western blotting, Transwell and BrdU assays. Seven miRNAs were significantly decreased in IPF lungs, with miR-130b-3p being the highest in the miRNA-mRNA network. Insulin-like growth factor (IGF-1) was a target gene of miR-130b-3p in the epithelium. miR-130b-3p inhibition in the epithelium induced collagen I expression and enhanced the proliferation and migration ability of fibroblast in co-culture systems, which mimicked the functions of exogenous IGF-1 on fibroblasts. Neutralizing IGF-1 with an antibody significantly reduced the modulatory effects of miR-130b-3p inhibitor-transfected epithelium on the activation of fibroblasts. Our results show that miR-130b-3p was downregulated in IPF lungs. miR-130b-3p downregulation contributed to the activation of fibroblasts and the dysregulated epithelial-mesenchymal crosstalk by promoting IGF-1 secretion from lung epithelium, suggesting a key regulatory role for this miRNA in preventing lung fibrosis.

## Introduction

Idiopathic pulmonary fibrosis (IPF) is a chronic, progressive, irreversible, and usually lethal lung disease characterized by unknown causes and few treatment options[1]. Physiologically, interactions between alveolar epithelial cells and fibroblasts can regulate lung development and homeostatic equilibrium. However, the abnormal epithelial-mesenchymal crosstalk will form a profibrotic milieu and hamper the normal alveolar wound-repair process. This is now believed to play a central role in the pathogenesis of IPF[2–4]. Repetitive or persistent injuries to the alveolar epithelium are the “prime mover” to initiate lung fibrosis. The injured epithelium will launch a dialogue with lung mesenchyme and the interactions between epithelial cell and fibroblast will eventually create a vicious cycle[5]. Many soluble factors classically including growth factors/cytokines, lipid mediators, and reactive gaseous molecules are involved in the regulation of epithelial-mesenchymal crosstalk. Transforming growth factor beta-1 (TGF-β1)[6], connective tissue growth factor (CTGF)[7], keratinocyte growth factor (KGF)[8], prostaglandin (PG)E_2_[9], and H_2_O_2_[10] have been implicated in these interactions in α-smooth muscle actin (SMA) gene expression, basement membrane formation, fibroblast proliferation and epithelial apoptosis.

MicroRNAs (miRNAs) are single-stranded, endogenous, and non-coding RNAs, approximately 20–25 nucleotides long in which a 7 nucleotides “seed region” existing in miRNAs is thought to be critical for binding target mRNA at complementary sites in 3’-untranslated regions (3’-UTR)[11]. Functionally, miRNAs act as a negative regulator of gene expression at a post-transcriptional level[12]. Previous miRNA microarrays showed that about 10% of the miRNAs was different between IPF and control lungs[13]. Furthermore, a comprehensive miRNAs analysis, performed in bleomycin-induced lung fibrosis, explored the relationship between miRNAs and apoptosis, Wnt signaling, Toll like receptor (TLR) signaling and TGF-β signaling pathway and showed that the miRNAs and the identified potential target genes may contribute to the understanding of the complex transcriptional program of lung fibrosis[14]. Therefore, several miRNAs were reported to play an important role in the pathogenesis of IPF[15]. However, the miRNA that contributes most to the disease evolution and the underlying modulatory mechanisms are still remained unknown and should be further elucidated.

In this report, we hypothesized that miRNAs might contribute to the abnormal epithelial-mesenchymal crosstalk though regulating soluble growth factor in lung epithelium. Hence, we analyzed the repertoire of miRNA expression in IPF lungs and found that miR-130b-3p was significantly downregulated. We then designed an *in vitro* cell study to show that miR-130b-3p played an important role in the abnormal epithelial-mesenchymal crosstalk by regulating insulin-like growth factor (IGF-1) expression in epithelium.

## Materials and Methods

### Lung tissues and microarray

Lung tissue was obtained from 4 IPF patients with histological evidence of usual interstitial pneumonia at the time of surgical lung biopsy or lung transplantation. The diagnosis of IPF was derived according to the standards accepted by the American Thoracic Society/European Respiratory Society. Histological normal lung tissues used as controls was obtained from 3 patients with primary spontaneous pneumothorax at the time of thoracoscopy with stapling of any air leak. All the patients were treated in Beijing Chao-Yang Hospital, Capital Medical University. Total RNA was isolated with Trizol, Biotinylated cDNA was prepared according to the standard Affymetrix protocol 3 from 250 ng total RNA by using Ambion^®^ WT Expression Kit. Following labeling, 5.5 μg of cDNA were hybridized for 16 hr at 45°C on GeneChip Human Transcriptome Array (Affymetrix, Santa Clara, CA) in Hybridization Oven 645. GeneChips were washed and stained in the Affymetrix Fluidics Station 450. GeneChips were scanned by using Affymetrix^®^ GeneChip Command Console (AGCC) which installed in GeneChip^®^ Scanner 3000 7G. The data were analyzed with Robust Multichip Analysis (RMA) algorithm using Affymetrix default analysis settings and global scaling as normalization method. Values presented are log2 RMA signal intensity.

This study was approved by the Ethics Committee of Beijing Chao-Yang Hospital of Capital Medical University, Beijing, China and written informed consent was obtained from all investigated subjects following the institutional guidelines.

### Bioinformatics analysis of differentially expressed microRNA

The gene ontology (GO) analysis was applied to predict the main functions of the target genes according to the gene ontology project[16,17]. Fisher’s exact test and χ^2^ were used to classify the GO category. And the false discovery Rate (FDR) was calculated to correct the *P* value. The smaller the FDR, the smaller the error in judging the *P* value. The standard difference screening was *P*<0.01.

Pathway analysis was used to find out the significant pathway of the differential genes according to the Kyoto Encyclopedia of Genes and Genomes (KEGG), Biocarta and Reatome[18,19]. Fisher’s exact test and χ^2^ were used to select the significant pathway, and the threshold of significance was defined by *P* value and FDR. The standard difference screening was *P*<0.05.

MiRNA-target gene network was established based on the GO and KEGG predicted data, for illustration of the relationship between miRNAs and their target genes. In the miRNA-mRNA network, the center of the network was represented by degree, the key miRNA and gene in the network always has the highest degree[20,21].

### Luciferase reporter assay

Human IGF-1 WT1 and WT2 3’-UTRs containing the putative binding sites of miR-130b-3p were amplified by PCR with primer (S1 Table), inserted into the firefly luciferase reporter vector pmiR-RB-REPORT^™^ (RibBio Co. Ltd) between the restrictive sites *Xho* I and *Not* I, and validated by sequencing. Their mutant constructs with a mutation of the miR-130b-3p seed sequence were generated with the mutagenic oligonucleotide primer (S1 Table), according to the manual of GeneTailor Site-Directed Mutagenesis System (Invitrogen Carlsbad, CA, USA). Cells were plated at 1.5×10^4^ cells/well on 96-well plates, and transfected with 100 ng/ml of either the wild-type or mutant 3’-UTR vector and 50 nM of either the miR-130b-3p mimic or miR-130b-3p Non-target Control (NC#24, Ribobio Co; Table 1) using lipofectamine 2000 (Invitrogen Carlsbad, CA, USA). At 48 hour after transfection, luciferase activity was measured in cell lysates using a dual-luciferase reporter kit (Promega, Madison, WI, USA). The *Renilla* luciferase signal was normalized to the firefly luciferase signal for each individual analysis.

**Table 1:** Oligonucleotide sets used for miRNA mimic, negative control and inhibitor.

Oligo Set	Sequences	Accession
miR-130b-3p mimic	5’-CAGUGCAAUGAUGAAAGGGCAU-3’	MIMAT0000691
NC (cel-miR-67-3p)	5’-UUUGUACUACACAAAAGUACUG-3’	MIMAT0000039
miR-130b-3p inhibitor	5’-GUCACGUUACUACUUUCCCGUA -3’	―

### Cell culture and ELISA for IGF-1 level in culture supernatant

The human adenocarcinoma A549 cells (CCL-185; ATCC, Rockefeller, MD, USA) were maintained in RPMI 1640 (Gibico, Grand Island, NYC, USA) containing 10% fetal bovine serum (FBS, Gibco, Grand Island, NYC, USA) and the lower passages were used[22]. Normal human primary type II alveolar epithelial (ATII) cells were maintained in the special alveolar epithelial cell media (Sciencell, San Diego, CA, USA) and the passages between 2 and 4 were used as previously described[23]. Human fetal lung fibroblast (MRC5, CCL-171^™^; ATCC, USA was cultured in alpha-MEM (Invitrogen Carlsbad, CA, USA) containing 10% FBS (Gibco, Grand Island, NYC, USA) and the lower passages were used[24]. All cells were incubated in 5% CO2 atmosphere at 37°C. A subconfluent culture of MRC5 was starved for 24 hours prior to stimulation with IGF-1 or co-culturing with epithelium. The modulatory effects of IGF-1 on MRC5 activities were investigated by incubating for different periods of time (48 and 72 hours) at two concentrations of IGF-1 (50 and 100 ng/ml; R&D Systems, Abingdon, Oxon, UK). A549 and ATII at 50% confluency in 6-well plates was transfected respectively with 50 nM miR-130b-3p mimic, negative control and inhibitor (Ribobio, Guangzhou, China; Table 1) using 1XtiboFECT CP buffer and reagent (Ribobio, Guangzhou, China). After 48 h, the IGF-1 in supernatant was measured by ELISA kit 1 (R&D Systems, Abingdon, UK). The modulatory effects of miRNA-transfected A549 or ATII on MRC5 activities were studied by using co-culture system. The modulatory mechanisms were determined by using human IGF-1 antibody (R&D Systems, Minneapolis, MN) added to the co-culture systems.

### RNA isolation and real-time PCR

miRNA quantifiation: Bulge-loop^™^ miRNA qRT-PCR Primer Sets (one RT primer and a pair of qPCR primers for each set) specific for miR-130b-3p are designed by RiboBio (Guangzhou, China). Briefly, the total RNA was extracted using Trizol reagent (Invitrogen, Carlsbad, CA). The cDNA was synthesized using the PrimeScript RT reagent Kit (TaKaRa, Tokyo, Japan) and real-time PCR was performed using the SYBR Premix Ex Taq (TaKaRa, Tokyo, Japan) on an ABI PRISM 7500 instrument (Applied Biosystems, Foster, CA, USA). The fold-change of the target genes was calculated using the 2^-△△CT^ method. mRNA quantification was performed similar as miRNA quantification, except that cDNA is reverse transcribed using oligo (dT)18 primer. The primers used are listed in S1 Table.

### Protein extraction and western blot

Protein from harvested cells was separated on a SDS-PAGE gel and then transferred onto polyvinylidene difluoride membranes (Millipore, USA). After blocking, the membranes were incubated overnight at 4°C with rabbit polyclonal anti-collagen I primary antibody (Abcam, Cambridge, CA, USA) and mouse polyclonal anti-β-actin primary antibody (California Bioscience, Coachella, CA, USA). The membranes were then treated with IRDye^ࡊ^800 (green) or IRDye^ࡊ^700 (red) conjugated affinity purified anti-rabbit or anti-mouse IgG (LI-COR, NE, USA). The positive western bands were visualized using a LI-COR Odyssey infrared double-fluorescence imaging system (LI-COR).

### Wound healing and migration assay

For the wound healing assay, cells were seeded in six-well plates and were subconfluent at the time scratch wounds were applied to each well. This was accomplished with a 200 μl pipette tip, and cell debris was washed off twice with PBS. Then Fresh medium, with or without IGF-1, was added to the wells and cells were incubated for up to 48 hours. Phase contrast light microscope pictures were taken on an inverted microscope immediately after scratch wounding (0 hour), and 24 hours and 48 hours. All experiments were conducted in duplicate and repeated three times.

The migration assay was performed by using Transwell inserts (8 μm pore size, Millipore, Watford, UK). Approximately 2 × 10^4^ cells in 200 μl of serum-free medium were placed in the upper chamber. 600 μl of medium with 10% FBS was supplemented in the lower chamber. For the co-culture experiments, miRNA-transfected A549 or ATII was seeded in the lower chamber in 600 μl of same medium containing 10% FBS. Non-migrating cells were removed by cotton swabbing and the migrating cells on the reverse side were stained with 0.1% crystal violet. Images of migrating cells were taken using a digital camera under a microscope at 4 × magnification.

### Bromodeoxyuridine (BrdU) assay

For the IGF-1 stimulation experiments, MRC5 was seeded on glass coverslips in 24-well plates. For the co-culture experiments, starved MRC5 was seeded on glass coverslips in the lower chamber, and miRNA-transfected A549 or ATII was seeded in the upper chamber. Cell proliferation was detected by BrdU assay (Roche Diagnostics GmbH, Germany). BrdU positive rate was evaluated by counting 5 different microscopic fields per time point.

### Statistical analysis

SPSS 16.0 (SPSS, Chicago, IL, USA) software was used for analyses. Data were reported as means ± SEM and analyzed using one-way analysis of variance followed by Neumann-Keuls multiple comparison test, *P* value < 0.05 was considered statistically significant. Statistical tests and graphs were done with GraphPad Prism 5.0 (GraphPad Inc., San Diego, CA).

### GEO accession numbers

The data obtained from miRNA sequencing studies were deposited in the Gene Expression Omnibus database at NCBI (http://www.ncbi.nlm.nih.gov/geo/). The accession number for GEO is GSE75647.

## Results

### MiRNAs were differentially expressed in IPF lungs and bioinformatics prediction revealed the role of miR-130b-3p in IPF

**Fig 1:**
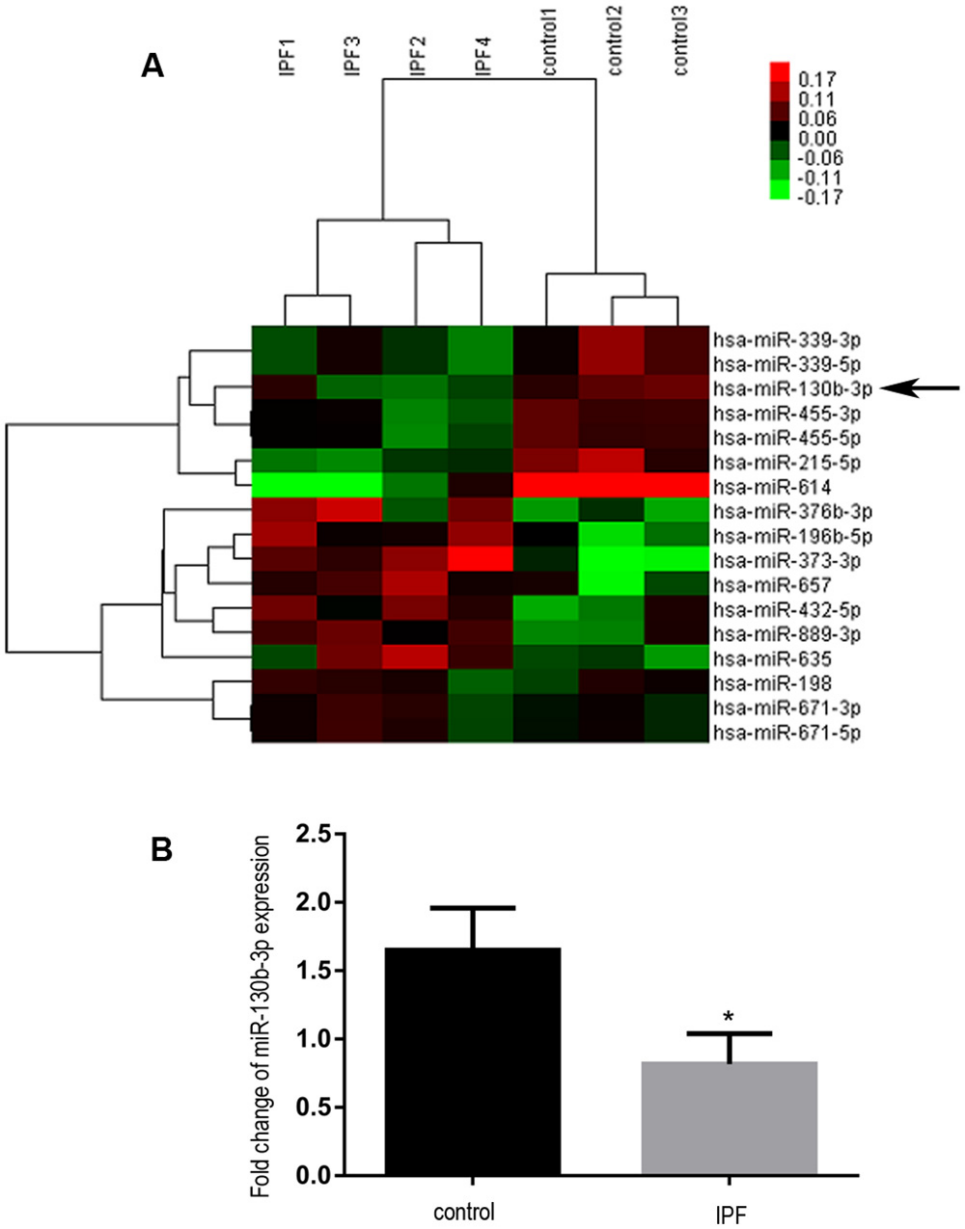
Downregulation of miR-130b-3p in IPF. (A) An unsupervised, hierarchical cluster heat map showed 17 differently expressed miRNAs between 4 IPF lungs and 3 normal lungs. Rows represent statistically significant (*P*<0.05) differentially expressed miRNAs, and columns represent patients and controls. Down-regulated miRNAs are shown in progressively brighter shades of green, depending on the fold difference. Up-regulated miRNAs are shown in progressively brighter shades of red. The names of dysregulated miRNAs are provided in the right of the heat map. An arrow is to denote miR-130b-3p. (B) Quantitative real-time polymerase chain reaction (qRT-PCR) confirmation of the microarray results demonstrated significant change of miR-130b-3p in lung tissues. *P<0.05.

To determine the expression of miRNAs in IPF, we analyzed 4 IPF lungs and 3 normal lungs on Affymetrix miRNA microarrays. As shown in the heat map representing an unsupervised, hierarchical cluster analysis of the two study groups (Fig 1A), 17 miRNAs were differentially expressed in IPF lungs, including 10 upregulated and 7 downregulated miRNAs. The array also identified published miRNAs with the same expression patterns. For example, miR-376b was significant upregulated expressed and consistent with previous dataset GSE27430[25]. The absence of some miRNAs like miR-21 and miR-155 was probably due to the limitation of sample number we used. Moreover, GO and KEGG were applied to predict the possible functions and pathways of the 17 miRNAs. The regulated GOs were related to cell proliferation, signal transduction, protein phosphorylation, apoptotic process, immune response and TGF-β signal pathway (Fig A in S1 File). The pathways predicted by KEGG analysis were mitogen activated protein kinase (MAPK), phosphatidyl inositol 3 kinase (PI3K)-AKT, extracellular matrix (ECM)-receptor interaction and adherens junction (Fig B in S1 File).

To determine the key differentially expressed miRNAs in IPF, the miRNA-target gene network was performed (Fig C in S1 File). We showed that the miR-130b-3p, one of downregulated miRNAs, was the highest-ranked miRNA in the network (Table 2). Furthermore, miR-130b-3p was also shown in another dataset (GSE27430)[25]. Therefore, it was selected to predicted potential targets and functions. Moreover, the downregulation of miR-130b-3p in IPF lung tissues was also validated by qRT-PCR (Fig 1B).

**Table 2:** Degree of differentially expressed microRNAs.

MicroRNA	MicroRNA style	Degree
has-miR-130b-3p	Downregulated	69
has-miR-373-3p	Upregulated	59
has-miR-889-3p	Upregulated	24
has-miR-196b-5p	Upregulated	18
has-miR-376b-3p	Upregulated	18
has-miR-455-3p	Downregulated	13
has-miR-198	Upregulated	12
has-miR-455-5p	Downregulated	11
has-miR-657	Upregulated	11
has-miR-432-5P	Upregulated	10
has-miR-671-5p	Upregulated	10
has-miR-215-5p	Downregulated	8
has-miR-339-5p	Downregulated	8
has-miR-635	Upregulated	8
has-miR-671-3p	Upregulated	5
has-miR-614	Downregulated	3
has-miR-339-3p	Downregulated	1

### IGF-1 was a target gene of miR-130b-3p

To find the target genes of miR-130b-3p, two algorithms (TargetScan and miRDB) were used for the prediction of the targets. Only the targets identified by both two algorithms were analyzed further. As shown in S1 File, 69 putative target mRNAs of miR-130b-3p were found and the putative target gene analysis indicated that miR-130b-3p may regulate IGF-1.

**Fig 2:**
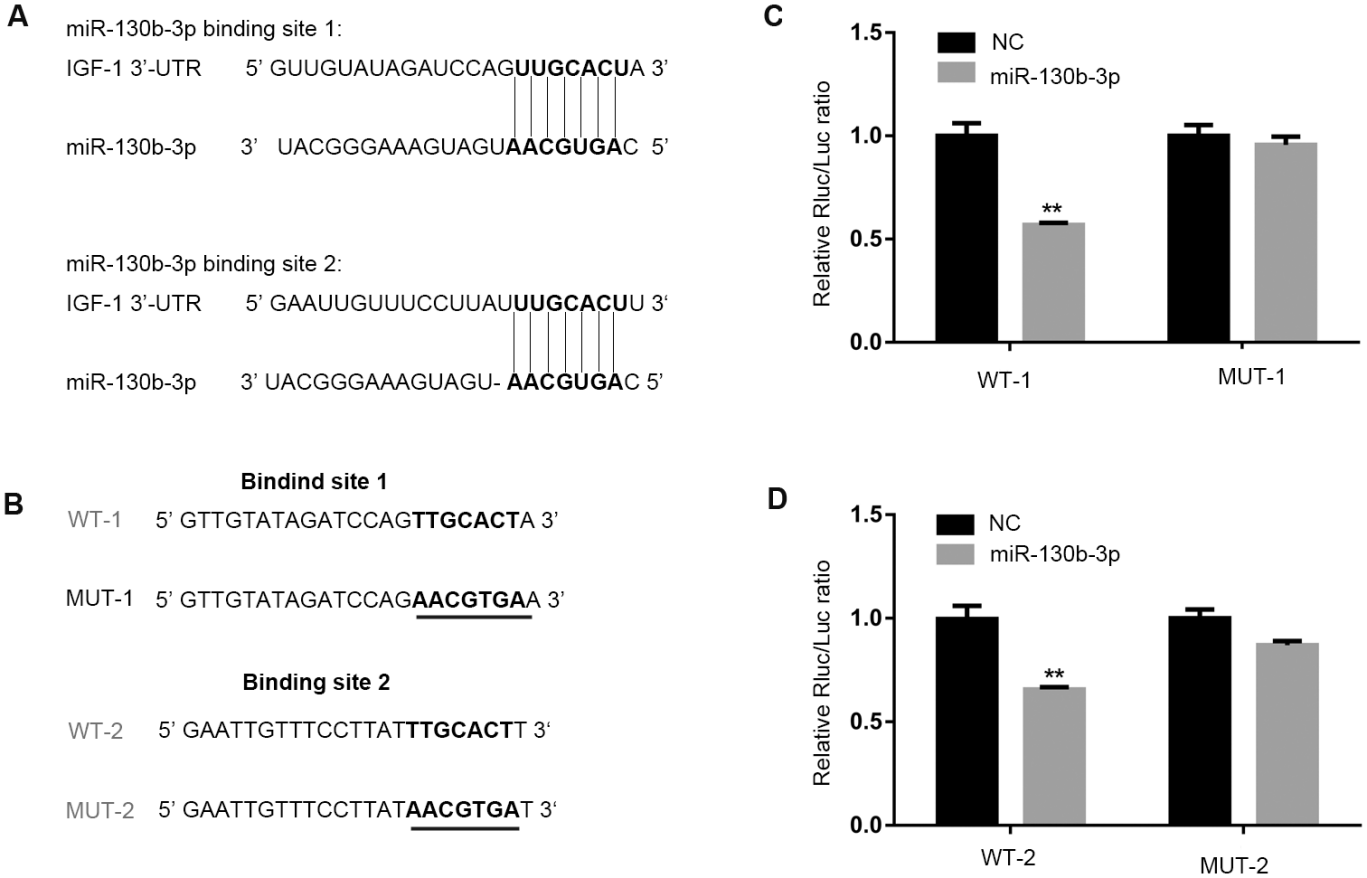
IGF-1 is a target gene of miR-130b-3p. (A) Predicted interaction between miR-130b-3p and the two putative binding sites in the IGF-1 3’-UTR. miR-130b-3p seed sequence is in bold. The representation is limited to the region around the miR-130b-3p complementary site. The number of region position is 367–374 (binding site 1) and 453–459 (binding site 2). (B) Generation by site-directed mutagenesis of human IGF-1 3’-UTR mutants in the putative binding sites. The mutated nucleotide is underlined, and the original nucleotide is its complementary one: MUT1 for the putative binding site 1, MUT2 for the putative binding site 2. Cells were plated at 1.5×10^4^ cells/well on 96-well plates, and cotransfected with 100 ng/ml of either the wild-type or mutant 3’-UTR vector of binding site 1 (C) or binding site 2 (D) and 50 nM of either the miR-130b-3p mimic or miR-130b-3p NC using lipofectamine 2000, At 48 h after transfection, The *Renilla* luciferase activity was measured and normalized to the firefly luciferase signal for each individual analysis. **P<0.01.

According to the TargetScan algorithm, sequence alignment of the miR-130b-3p complementary site in the 3’-UTR of IGF-1 mRNA allowed us to identify two conserved binding sites, position 367–374 and position 453–459, respectively (Fig 2A). To further determine whether miR-130b-3p targeted IGF-1 directly by 3’-UTR interaction, we cloned two fragments of the human IGF-1 3’-UTR mRNA containing the two putative miRNA-binding sites into the luciferase reporter vectors and transfected them into cells in the presence of either miR-130b-3p mimic or control. As anticipated, it showed that miR-130b-3p efficiently inhibited luciferase activity, whereas the inhibition was not observed when control miRNA was used (Fig 2C and 2D; S2 Table). Correspondingly, we also generated two mutant reporters in which the seed binding sequence in 3’-UTR of IGF-1 was mutated (Fig 2B), and the luciferase activity of the two mutant reporters was not affected by miR-130b-3p (Fig 2C and 2D; S2 Table). Collectively, we showed that miR-130b-3p was able to target IGF-1 with two binding sites in its 3’-UTR.

### miR-130b-3p modulated IGF-1 expression in alveolar epithelial cells

**Fig 3:**
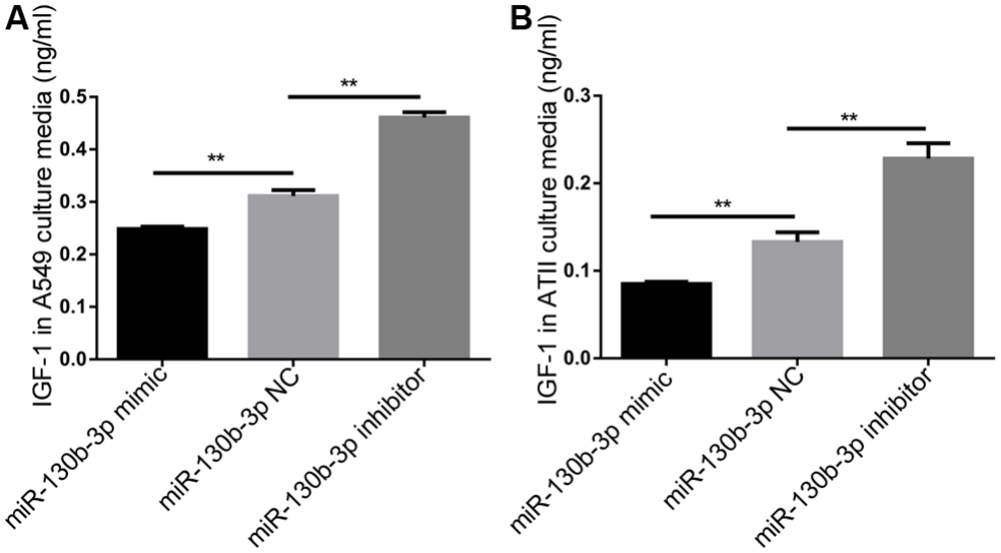
miR-130b-3p was a negative regulator of IGF-1 expression. A549 (A) or ATII (B) was transfected with 50 nM miR130b-3p mimic, negative control and miR130b-3p inhibitor. 48 h after transfection, the protein levels of IGF-1 in the supernatants were then determined by ELISA. The experiments were repeated three times. **P<0.01.

Both mRNA and protein level of IGF-1 were upregulated in IPF lungs as reported in previous studies[26,27,28]. To illustrate a potential relationship between downregulation of miR130b-3p and concomitant upregulation of IGF-1 in IPF lungs we transfected miR-130b-3p mimic, negative control (NC), and inhibitor (Table 1) into A549 or ATII. 48 hours after transfection, an ELISA assay was performed to determine the protein level of IGF-1 in the supernatants. We showed that miR-130b-3p inhibitor upregulated, whereas miR-130b-3p mimic downregulated the expression of IGF-1 in both A549 (Fig 3A and S3 Table) and ATII (Fig 3B and S3 Table) compared with NC.

### Exogenous IGF-1 exerted a profibrotic effect on human lung fibroblasts

**Fig 4:**
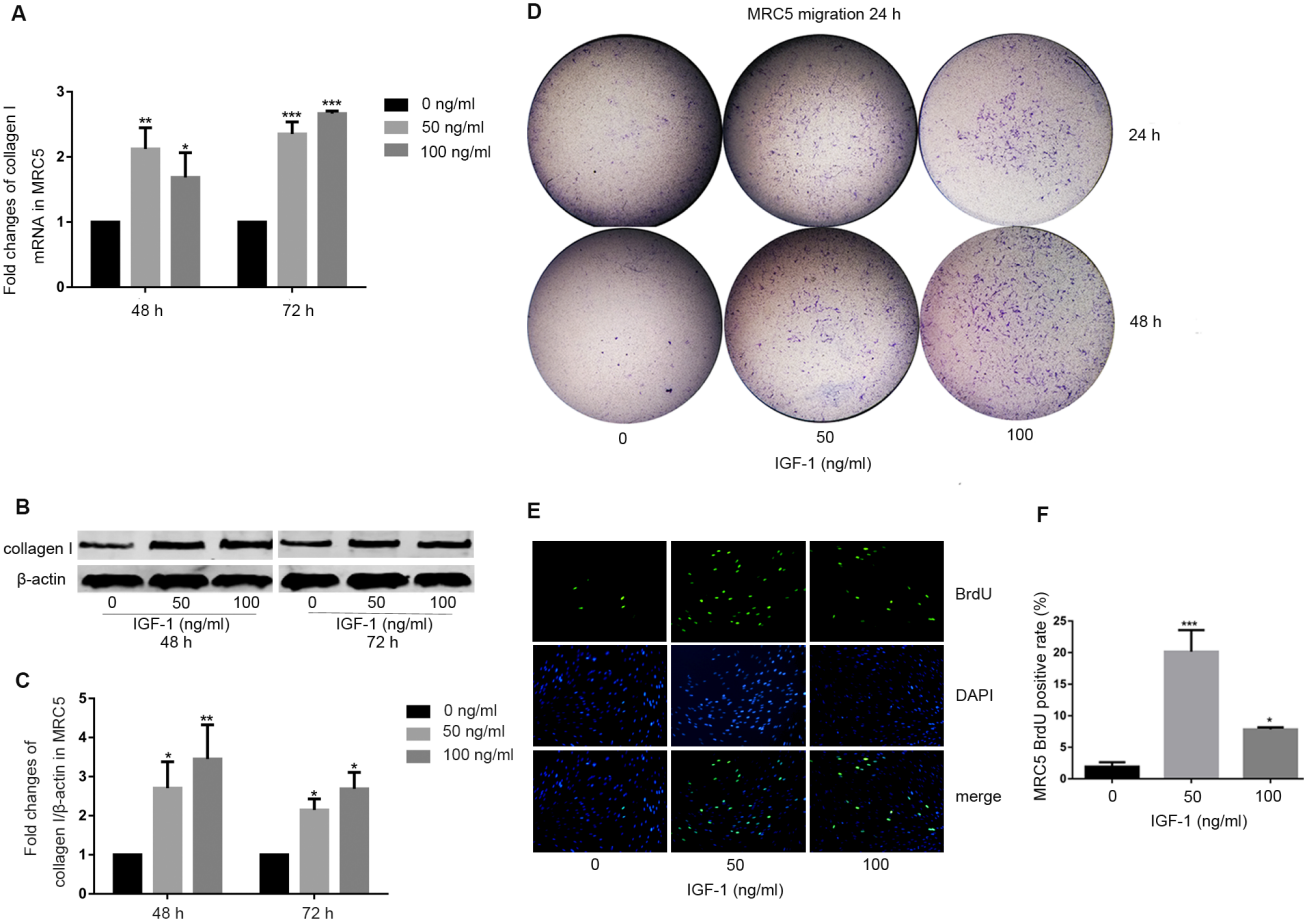
regulated the activation of MRC5 human primary fibroblasts. (A-C) IGF-1 induced collagen I expression of MRC5. MRC5 was treated with or without IGF-1 for 48 or 72 hours. (A) Collagen I mRNA level was measured with real-time PCR. (B and C) Collagen I protein level was determined by western blot analysis and normalized to β-actin. (D) IGF-1 enhanced migration ability of MRC5. MRC5 was treated with or without IGF-1 for 24 or 48 hours. Then MRC5 was harvested, and transwell migration assay was used to assess the 24 hours migration ability of MRC5. (E and F) IGF-1 enhanced proliferation ability of MRC5. (E) MRC5 was treated with IGF-1 for 24 hours, the proliferation of MRC5 was determined by immunofluorescent staining with anti-BrdU (green), and nuclei were stained with DAPI (blue). Original magnification, 200×(E). (F) Quantification of proliferating cells, the chart represents the percentage of BrdU positive cells among the total MRC5. All experiments were performed in triplicate. *P<0.05. **P<0.01, ***P<0.001.

Next, we asked whether the exogenous IGF-1 could exert a profibrotic effect on human lung fibroblasts. We treated human fetal lung fibroblast MRC5 with IGF-1 (50 and 100 ng/ml) for indicated time and analyzed the expression of collagen I. We found that IGF-1 increased collagen I expression both at mRNA level (Fig 4A and S4 Table) and protein level (Fig 4B and 4C; Fig A in S2 File and S5 Table) in a dose-dependent manner.

To determine whether IGF-1 could act as a chemotactic factor, we validated the migration of MRC5 and found a marked elevation in migration ability in IGF-1-treated MRC5 (Fig 4D and S1 Fig). Furthermore, to analyze the effect of IGF-1 on MRC5 proliferation, the BrdU assay was then performed and demonstrated that both doses of IGF-1 stimulation (50 and 100 ng/ml) significantly potentiated the proliferation of MRC5 cells (Fig 4E). However, IGF-1 (50 ng/ml) was more effective than IGF-1 (100 ng/ml) in promoting MRC5 proliferation indicated by a higher BrdU positive rate (Fig 4F and S6 Table).

### miR-130b-3p inhibition in epithelial cells increased collagen I expression of fibroblasts in co-culture systems

**Fig 5:**
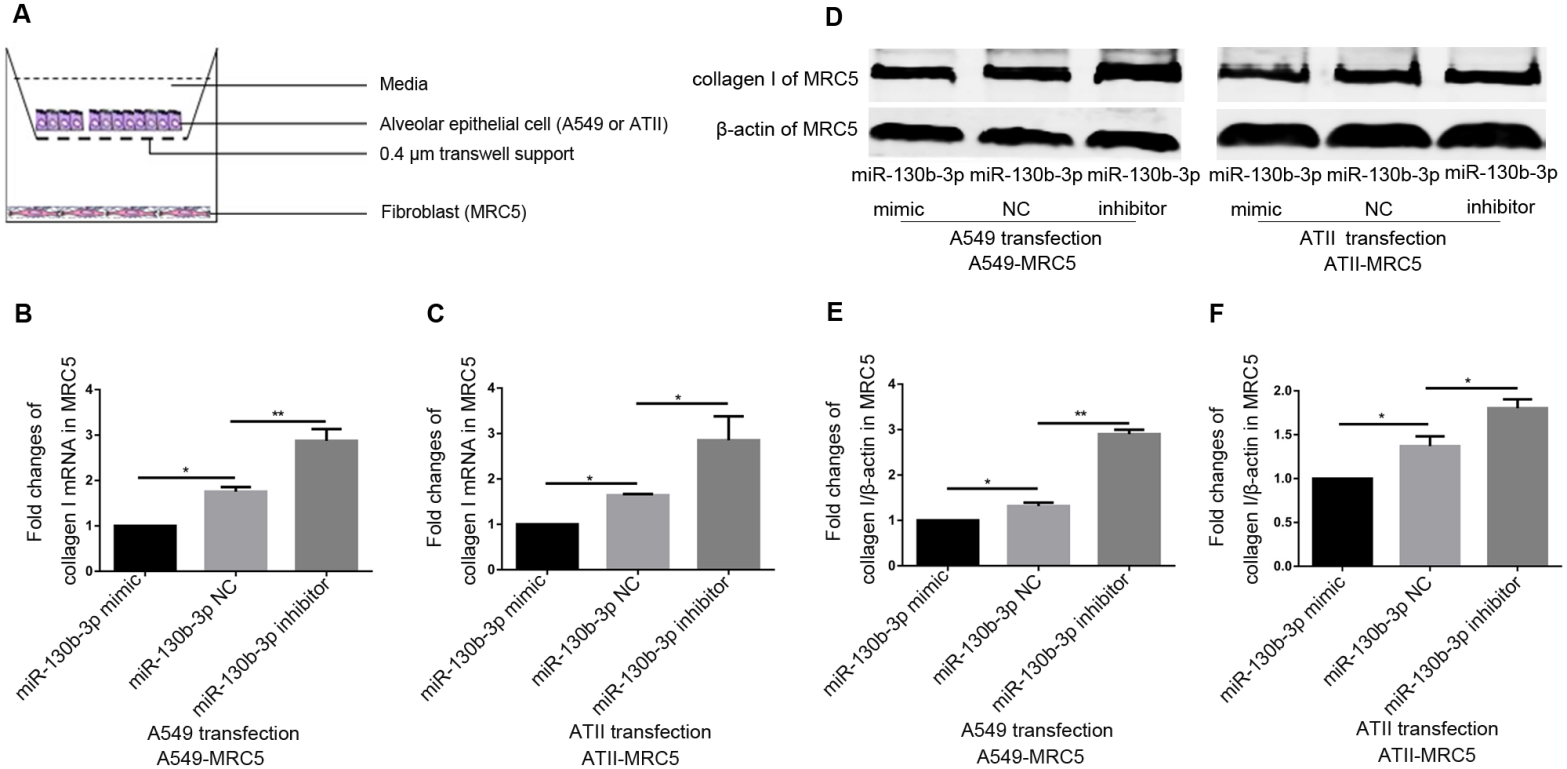
miR-130b-3p inhibition in epithelial cell induced collagen I expression of MRC5 in co-culture system. (A) Epithelial cell-fibroblast co-culture system (0.4 μm) was established to detect the collagen I expression of MRC5 in 6-well plates, where epithelial cells were seeded in the upper chamber, and fibroblasts were seeded in the lower chamber. A549 or ATII was transfected with 50 nM miR-130b-3p mimic, negative control and miR-130b-3p inhibitor. 24 hours after transfection, epithelial cells were harvested and co-cultured with MRC5, after 48 hours, real-time PCR was performed to detect the collagen I mRNA level of MRC5 in A549 (B) and ATII (C) co-culture systems. (D) Western blot analysis for collagen I protein level of MRC5 in co-culture systems. Relative collagen I protein levels of MRC5 in A549 (E) and ATII (F) co-culture systems were normalized to β-actin. *P<0.05, **P<0.01.

Considering the fact that miR-130b-3p negatively regulated IGF-1 expression in epithelial cells, we then determined the role of miR-130b-3p in epithelial cell-fibroblast crosstalk. To accomplish this, we established a co-culture system of alveolar epithelial cells with fibroblasts (Fig 5A). miRNA-transfected A549 or ATII was cocultured with MRC5 for 48 hours, then the collagen I expression of MRC5 was determined. We showed that miR-130b-3p inhibition in A549 or ATII increased MRC5 both mRNA (Fig 5B and 5C; S7 Table) and protein level (Fig 5D–5F; Fig B in S2 File and S8 Table) of collagen I in co-culture systems.

### miR-130b-3p inhibition in epithelial cells enhanced the proliferation and migration ability of fibroblasts in co-culture systems

**Fig 6:**
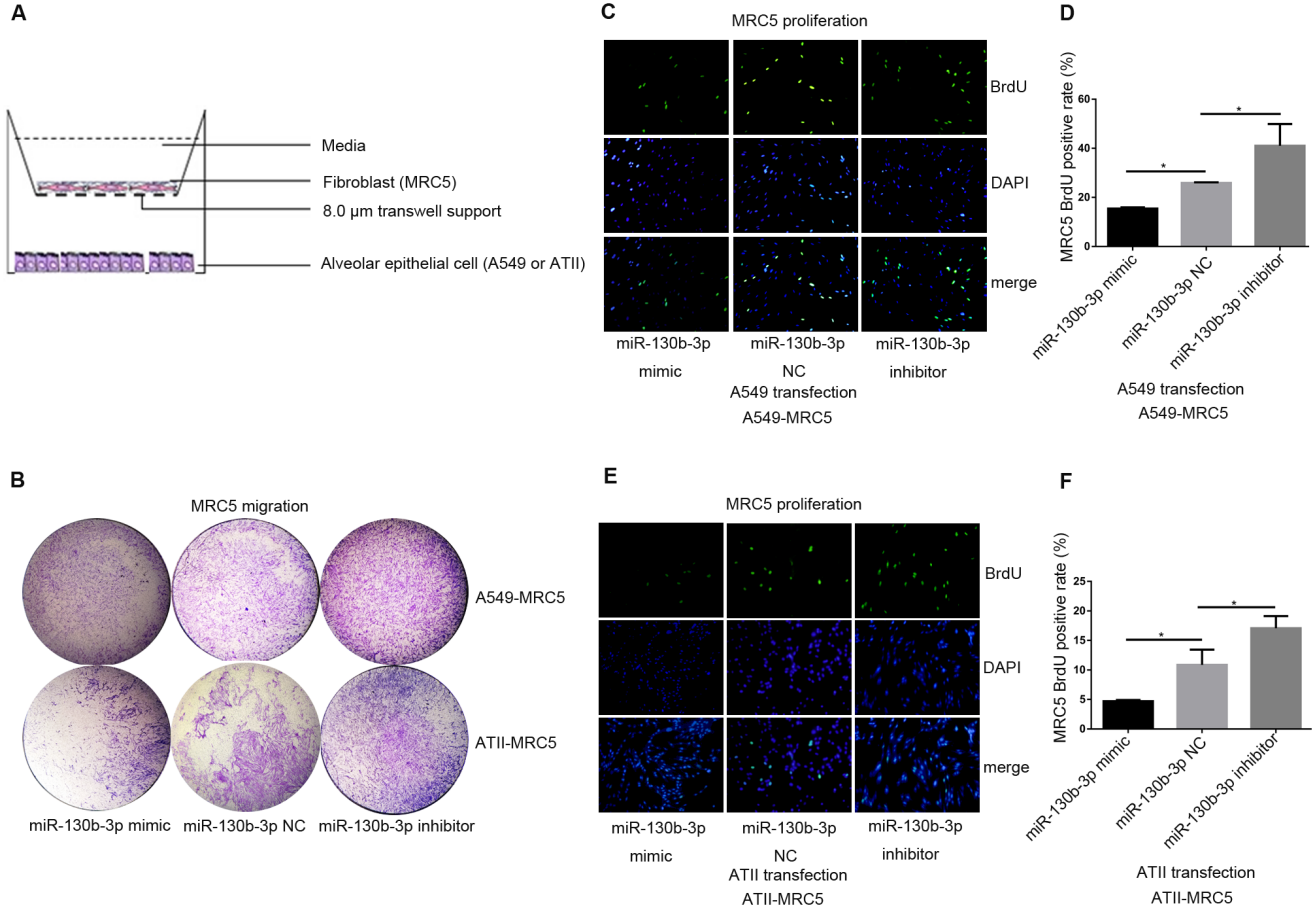
miR-130b-3p inhibition in epithelial cell enhanced fibroblast migration and proliferation in co-culture systems. (A) Another epithelial cell-fibroblast transwell co-culture system (8.0 μm) was constructed to determine the migration ability of MRC5 in co-culture system, where epithelial cells were seeded in the lower chamber, fibroblasts were seeded in the upper chamber, (B) A549 or ATII was transfected with 50 nM miR-130b-3p mimic, negative control and miR-130b-3p inhibitor, 24 hours after transfection, epithelial cells were harvested and co-cultured with starved MRC5. After 48 hours, transwell migration assay was used to assess the migration ability of MRC5. (C-F) All cells were performed as described in B. Co-culture system (0.4 μm) was used, where epithelial cells were seeded in the upper chamber, and fibroblasts were seeded in the lower chamber. 24 hours later, BrdU assay was preformed to assess the proliferation ability of MRC5 co-cultured with A549 (C and D) or ATII (D and F). Original magnification, 200×(C and E). The experiments were repeated three times. *P<0.05.

In order to analyze the relationship between the miR-130b-3p level of epithelial cells and the migration ability of MRC5, a transwell co-culture system was established (Fig 6A). Fig 6B showed that MRC5 migration ability was significantly elevated in the presence of A549 or ATII transfected with miR-130b-3p inhibitor. In contrast, only a few migration cells were seen in the miR-130b-3p mimic co-culture system compared to the control system. Furthermore, the proliferation ability of MRC5 in response to miRNA-transfected epithelial cells was also determined by using another co-culture system (Fig 5A). We showed that inhibition of miR-130b-3p enhanced, whereas overexpression of miR-130b-3p in A549 (Fig 6C and 6D; S9 Table) or ATII (Fig 6E and 6F; S9 Table) attenuated the proliferation ability of MRC5. Taken together, inhibition of miR-130b-3p in epithelial cells enhanced both the proliferation and migration ability of MRC5 in co-culture systems.

### Human IGF-1 antibody prevented miR-130b-3p inhibitor-induced profibrotic effects in fibroblasts

**Fig 7:**
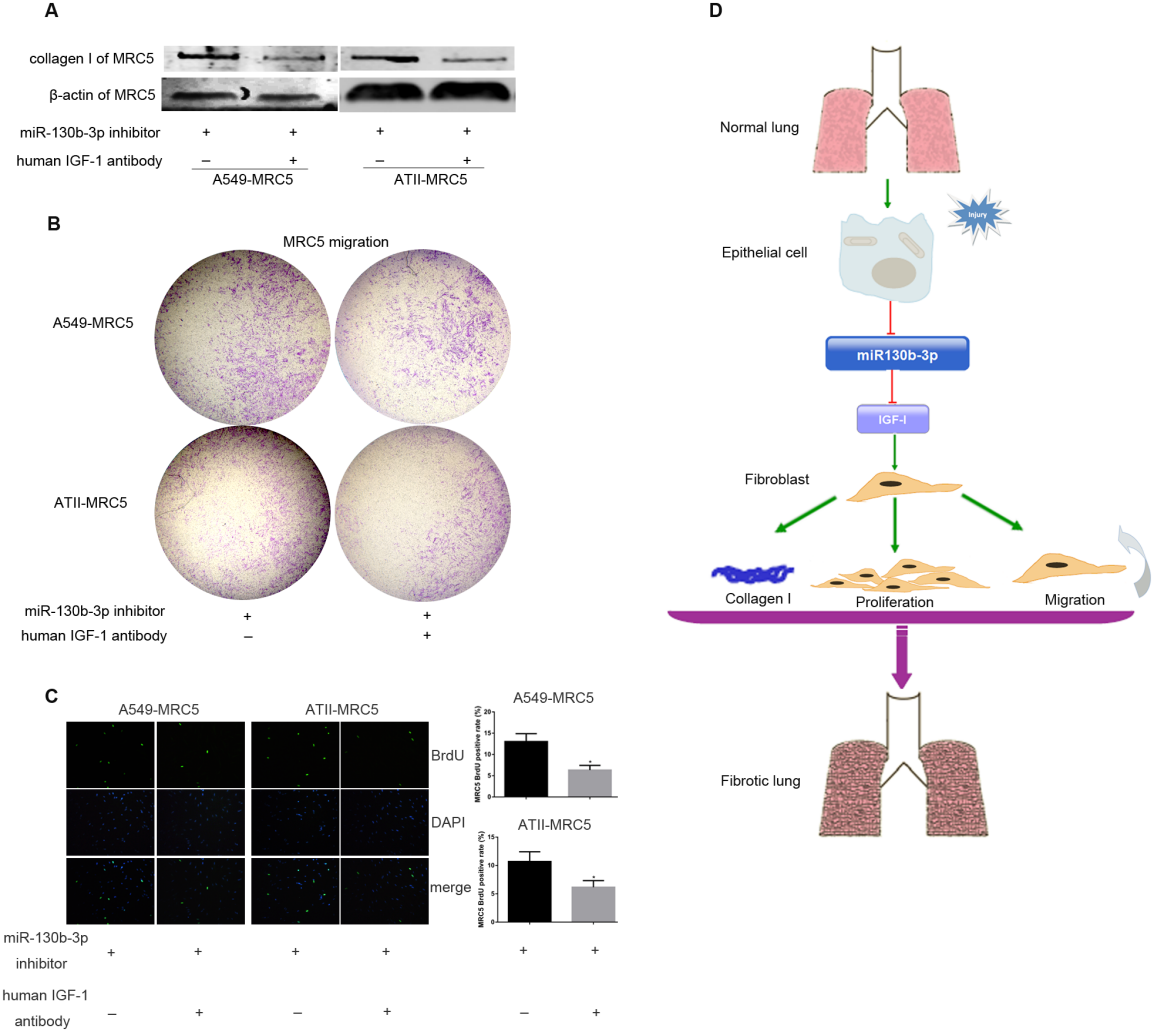
Human IGF-1 antibody prevented miR-130b-3p inhibitor-induced profibrotic effects on fibroblasts. A549 or ATII was transfected with 50 nM miR-130b-3p inhibitor, 24 hours after transfection, A549 or ATII was harvested and co-cultured with starved MRC5 with or without human IGF-1 antibody using 0.4 μm co-culture system (A and C) or 8.0 μm co-culture system (B). After 48 hours, the collagen I protein level (A), migration (B), and proliferation (C) of MRC5 was determined. Original magnification, 200×(C). (D) Persistent injury of alveolar epithelial cells caused decreased expression of miR-130b-3p, which in turn failed to depress the expression of IGF-1. IGF-1, acting as a paracrine activator, regulated the activation of fibroblast though miR-130b-3p-dependent mechanisms. Altogether, the downregulation of miR-130b-3p in lung may contribute to the development of lung fibrosis. *P<0.05.

We finally explored whether miR-130b-3p inhibition in A549 or ATII affected MRC-5 activities by promoting IGF-1 expression. We therefore cocultured miR-130b-3p inhibitor-transfected A549 or ATII and MRC5 with or without human IGF-1 antibody. We showed that collagen I expression (Fig 7A and Fig C in S2 File), migration (Fig 7B) and proliferation (Fig 7C and S10 Table) of MRC5 induced by miR-130b-3p inhibitor were significantly reduced by human IGF-1 antibody treatment. Altogether, these data suggested that miR-130b-3p exerted its role on MRC5 activities by directly targeting the expression of IGF-1 in A549 or ATII (Fig 7D).

## Discussion

In this study, we explored the expression, regulation, and potential role of miRNAs in IPF. We focused on miR-130b-3p, one of the downregulated miRNAs, and found that miR-130b-3p played an important role in the epithelial-mesenchymal crosstalk. miR-130b-3p modulates fibroblast activities by directly targeting the 3’-UTR of IGF-1 mRNA and downregulating IGF-1 expression in epithelial cells.

It is believed that the abnormal epithelial-mesenchymal crosstalk lead to the formation of profibrotic milieu and impaired alveolar repair in fibrotic lung diseases[2]. miRNAs regulate lung fibrosis in many ways, including epithelial mesenchymal transition[13,29], ECM synthesis[30], fibroblast activation and proliferation[31,32], and TGF-β signal pathway[33,34]. Interestingly, emerging evidence demonstrated that miRNAs also contribute to the epithelial-mesenchymal crosstalk. miR-155[35] and miR-17-92 cluster (miR-18a, 19a/b)[36,37] seemed to be essential for mediating the expression of soluble growth factor including KGF and CTGF. However, the exact relationship between miRNAs and the epithelial-mesenchymal crosstalk in lung fibrosis still remains elusive.

miRNA expression profiling has provided important new insights of many human diseases including tissue fibrosis by using high-throughput genomic approaches[38–40], which has been previously successfully applied to IPF, showing that miR-21 and let-7d were the important contributors to the disease process[13,31]. In this present study, we used 4 IPF lungs and 3 normal lungs to confirm the expression profile of miRNAs and showed that there were 17 significant modifications of lung miRNAs, including 10 upregulated and 7 downregulated miRNAs. Based on these novel findings, we speculate that the significant changes in the profile of miRNAs expression exert an important effect on the process of IPF.

miRNAs can act as master regulators of complex biological processes by mediating the expression of multiple genes[14]. Thus, the illustration of the critical genes and relevant pathways/network regulated by miRNAs is essential to understand the mechanisms underlying diseases. In our study, two bioinformatics analysis (GO and KEGG) were used to predict the major functions and pathways of the 17 dysregulated miRNAs. Moreover, miRNA-target gene network was also analyzed to predict their target genes according to GO and KEGG. We focused our study on miR-130b-3p because it was predicted to own the biggest degree in the miRNA-target gene network which suggested miR-130b-3p was the key miRNA.

Subsequent studies have found that miR-130b-3p could target IGF-1, which has been reported about its elevation in fibrotic lung diseases, such as IPF[26,27], coal worker’s lung fibrosis[40], fibroproliferative ARDS[41], as well as bleomycin-induced mouse lung fibrosis[42]. Additionally, previous studies showed that lung macrophages and epithelium of IPF patients expressed high level of IGF-1[26,28], and IGF-1 was putatively regulated by miRNAs in bleomycin-induced mouse lung fibrosis[43]. Therefore, we hypothesized that IGF-1, by its two binding sites in 3’-UTR, was the most important target gene of miR-130b-3p. miRNAs bind to the specific sequence in 3’-UTR of target mRNA and lead to mRNA degradation or translational repression[12]. In our study, a luciferase assay was performed to prove that miR-130b-3p targeted IGF-1 mRNA directly through a 3’-UTR interaction in the two binding sites. Furthermore, miR-130b-3p acted as a negative regulator of IGF-1 expression in A549 or ATII. Because A549 cells are transformed, immortalized cancer cell line, we also performed the parallel experiments with primary ATII cells. We observed an almost identical pattern of IGF-1 expression following transfection with miR-130b-3p in A549 and ATII. These results establish that the effect of miR-130b-3p on IGF-1 expression is not limited to transformed type II AECs, but can also be induced in normal human primary type II alveolar epithelial cells *in vitro*. Our data may provide new explanation about the adverse level of miR-130b-3p and IGF-1 *in vivo*.

Indeed, IGF-1 was reported to have broad physiologic functions including the stimulation of cell migration, growth, and inhibition of apoptosis by activating Insulin-like growth factor-1 receptor (IGF1R)[27]. In the current study, we showed that exogenous IGF-1 was able to stimulate collagen I synthesis, and enhance the migration and proliferation ability of lung fibroblast, which could be supported by previous reports[42,44]. IGF-1 is known to regulate miR-1 expression in different cell types[45,46]. Future studies are warranted to elucidate the role of miR-130b-3p-IGF-1-miR-1 axis in lung fibrosis. Altogether, IGF-1, a paracrine fibroblast activator produced by epithelial cells, played an important role in the activation of fibroblast. However, the upstream mechanisms modulating endogenous IGF-1 expression in the epithelial-mesenchymal crosstalk is still poorly understood.

Recently, emphasis on epithelial-mesenchymal crosstalk in IPF has shifted from the notion that IPF is an inflammatory disorder to another hypothesis that IPF is a disorder of alveolar injury and aberrant repair[47]. Indeed, many previous findings provided evidence that the injury and aberrant repair of alveolar epithelium can trigger the aberrant epithelial-mesenchymal crosstalk leading to the development of fibrosis[48–50]. In this study, to evaluate the effect of alveolar epithelium on lung fibroblasts *in vitro*, we constructed two epithelial cell-fibroblast co-culture systems, which would simulate conditions *in vivo* and allow for analysis of regulators and soluble growth factors. The co-culture systems may prove to be useful for the future studies of mechanisms involving in the epithelial-mesenchymal crosstalk.

Next, we determined the role of miR-130b-3p in the epithelial-mesenchymal crosstalk. Previous reports indicated that the fibroblasts were activated in the lung and released excessive collagen[51] and migration of fibroblasts into intra-alveolar spaces with subsequent deposition of extracellular matrix proteins was seen in lung fibrosis[42]. Furthermore, fibroblast proliferation was believed to be one of the critical features of lung fibrosis[52,53]. In our study, we found that miR-130b-3p inhibition in the epithelium was able to stimulate collagen I synthesis, enhance the migration and proliferation ability of fibroblasts in co-culture systems. These findings suggested that miRNA-transfected epithelium secreted different level of paracrine factors capable of influencing the activation of fibroblast. Notably, neutralization of IGF-1 significantly reduced the profibrotic effect of miR-130b-3p inhibition on fibroblast in co-culture systems. In different ways, our experiments altogether provide solid evidence that miR-130b-3p indeed plays an essential role in lung fibrosis and this role is indicated by mediating the abnormal epithelial-mesenchymal crosstalk at least in part, by modulating IGF-1 expression. These experimental results, along with previous study[54], support that in addition to targeting IGF-1, miR-130b-3p was also predicted to regulate some other important target genes, such as transforming growth factor beta receptor II (TGFBR2) and Wnt. Both of which are key regulators in tissue fibrosis including the differentiation of fibroblast to myofibroblast[1,55,56]. Further studies are needed to identify the role of miR-130b-3p on these target genes.

In summary, we showed that IPF lungs were different from normal lungs in their miRNA repertoire. Specifically, we used an *in vitro* co-culture study to validate the role of miRNA involvement in the abnormal epithelial-mesenchymal crosstalk in lung fibrosis. We believe that these findings have an important implication in our understanding of the molecular mechanism underlying IPF. Further investigation of miR-130b-3p-IGF1 axis would provide a new therapeutic intervention for this lethal fibrotic lung disease.

## Supporting Information

S1 Fig Wound healing show IGF-1 with a modulatory effect on MRC5 cell’s mobility. Serum starved MRC5 were incubated for 24 hours and 48 hours in the absence or presence of IGF-1 in alpha-MEM containing 0.1% FBS. (A) Wound healing assay was used to indirectly measure the migratory capacity of MRC5 in six-well plates. Time points 0, 24, 48 hours were observed at 40× magnification. (B) Summary of wound assay results. The average healing distances were measured at three different points per group with the changes of time. *P<0.05, *P<0.01.
(TIF)

S1 File GO and KEGG pathway analysis, and miRNA-target gene network analysis. (A) miRNA-GO network is based on the relationship of significant functions and miRNAs. The blue circle represents GOs and the suqure represents miRNAs. Straight line represents miRNA-GO relationship. (B) miRNA-KEGG analysis is established according to the pathway regulated by downregulated genes (up) and upregulated genes (below), the vertical axis is the pathway category and the horizontal axis is the (-LgP), the higher (-LgP) value, namely, the lower P value, represents that pathway has more important role in IPF. (C) miRNA-target-network: the red squares represent upregulated miRNAs and the blue squares represent downregulated miRNAs, the blue circles represent target genes, straight line represents miRNA-gene relationship. The degree (size) of the red or blue squares correlates the regulatory functionality of miRNAs. The bigger the degree, the more functions of the miRNA.
(RAR)

S2 File The original uncropped and unadjusted blots in Figs 4, 5 and 7. (A) The original blots in Fig 4B. (B) The original blots in Fig 5D. (C) The original blots in Fig 7A.
(TIF)

S1 Table Primer sequences (5’-3’) on the products of the PCR and RT-qPCR assay. (DOC)

S2 Table The data points underlying the graphs in Fig 2C and 2D. (DOC)

S3 Table The data points underlying the graphs in Fig 3A and 3B. (DOC)

S4 Table The data points underlying the graph in Fig 4A. (DOC)

S5 Table The data points underlying the graph in Fig 4C. (DOC)

S6 Table The data points underlying the graph in Fig 4F. (DOC)

S7 Table The data points underlying the graphs in Fig 5B and 5C. (DOC)

S8 Table The data points underlying the graphs in Fig 5E and 5F. (DOC)

S9 Table The data points underlying the graphs in Fig 6D and 6F. (DOC)

S10 Table The data points underlying the graphs in Fig 7C. (DOC)
