# Expression of Concern: miR-130b-3p Modulates Epithelial-Mesenchymal Crosstalk in Lung Fibrosis by Targeting IGF-1

**DOI:** 10.1371/journal.pone.0263701

**Published:** 2022-02-03

**Authors:** The PLOS ONE Editors

Following the publication of this article [1], the authors contacted the journal to state that an error occurred during figure preparation in Fig 7A and S2 File part C. Further concerns were subsequently raised by the editorial team and the authors regarding results presented in Fig 4 and Supporting Information S2–S10 Tables. Specifically,

- In Fig 4B and S2 File part A, the following data appear similar despite being used to represent different experimental results:
  ◦ The Fig 4B 48 h collagen I panel and the S2 File part A 72 h collagen I panel.
  ◦ The Fig 4B 48 h β-actin panel and the S2 File part A 72 h β-actin panel.
  ◦ The Fig 4B 72 h β-actin panel and the S2 File part A 48 h β-actin panel.
- In Fig 4B, the 72 h collagen I panel does not appear to match the 72 h collagen I panel in S2 File part A, despite being used to represent the same original experiment.
- In Fig 5D, lane 1 of the β-actin of MRC5 panel for the ATII-MRC5 experiment is identical to lane 2 of the β-actin of MRC5 panel for the ATII-MRC5 experiment in Fig 7A. This identical band is also present in the corresponding panels in S2 File parts B and C.
- Contradictory to the figure legend, the blots presented in S2 File appear to be cropped blots.
- Quantitative data files in the Supporting Information tables (S2–S10 Tables) include summary data only, not the data points underlying the graphs, as indicated in the legends.

The authors commented that the issues in Fig 4B and S2 File part A were due to errors made during figure preparation. They stated that the 48 h and 72 h labels were incorrectly swapped in S2 File part A, and that the incorrect 72 h collagen I panel was included in Fig 4B. Updated versions of Fig 4 and S2 File are provided with this notice in which these errors have been addressed. The legend for S2 File has also been updated to indicate that the blots are cropped.

In relation to the issues highlighted by the authors in Figs 5D and 7A, they noted that the wrong image was used in Fig 7A to represent the β-actin of MRC5 panel for the ATII-MRC5 experiment. Updated versions of Fig 7 and S2 File are provided here. The authors stated that the updated β-actin of MRC5 panel includes the correct data from the original experiment.

The authors submitted image data to support the western blots presented in Figs 4B, 5D and 7A (S3, S4 and S5 Files). The authors stated that the membranes were cut prior to incubation with antibodies and some adjustments were made during image capture. Additionally, irregularities were identified in the background of the β-actin image provided for Fig 5D. Unadjusted raw images showing the full blot area are not available, and some of the images in S3–S5 Files are of low resolution. Therefore, the images provided do not fully resolve the concerns.

The available quantitative data underlying the results presented in this article are provided in the S6 File below. The authors stated that the raw data underlying Figs 5B and 5C are no longer available. Additionally, only processed data (normalized to controls) were provided for Figs 4A and 4C, 5E and 5F. Therefore, the article does not comply in full with the PLOS Data Availability policy.

Furthermore, the authors notified PLOS of the below errors in the Supporting Information tables. Updated Supporting Information tables in which these issues have been addressed are provided with this notice.

- In S2 Table, the values were labelled as means ± standard error of the mean (SEM), but the values in the table were means ± standard deviation.
- In S3 Table, the ‘A549’ column contained data that were derived from ATII cells, and the ‘ATII’ column contained data that were derived from A549 cells.
- In S4 Table, the value for 48 h 100 ng/ml was incorrect.
- In S5 Table, the value for 48 h 50 ng/ml was incorrect.
- In S6 Table, the value for 50 ng/ml was incorrect.
- S7 and S8 Tables were mislabelled: S7 Table should have been labelled S8 Table as the data corresponded to the graphs in Figs 5E and 5F, and S8 Table should have been labelled S7 Table as the data corresponded to the graphs in Figs 5B and 5C.
- In S7 Table in [1] (renamed S8 Table in this notice), values for NC for ATII and inhibitor for ATII were incorrect.
- In S10 Table, the value for Human IGF-1 antibody for ATII was incorrect.

The updated tables were reviewed by the *PLOS ONE* editorial team, and it was concluded that the errors did not impact the results presented in the corresponding graphs. Additionally, as S2–S10 Tables contain summary data and not data points underlying the graphs, the table legends and titles have been updated.

The *PLOS ONE* Editors issue this Expression of Concern to notify readers of the above concerns and relay the supporting data and updated figures provided by the authors.

**Fig 4:**
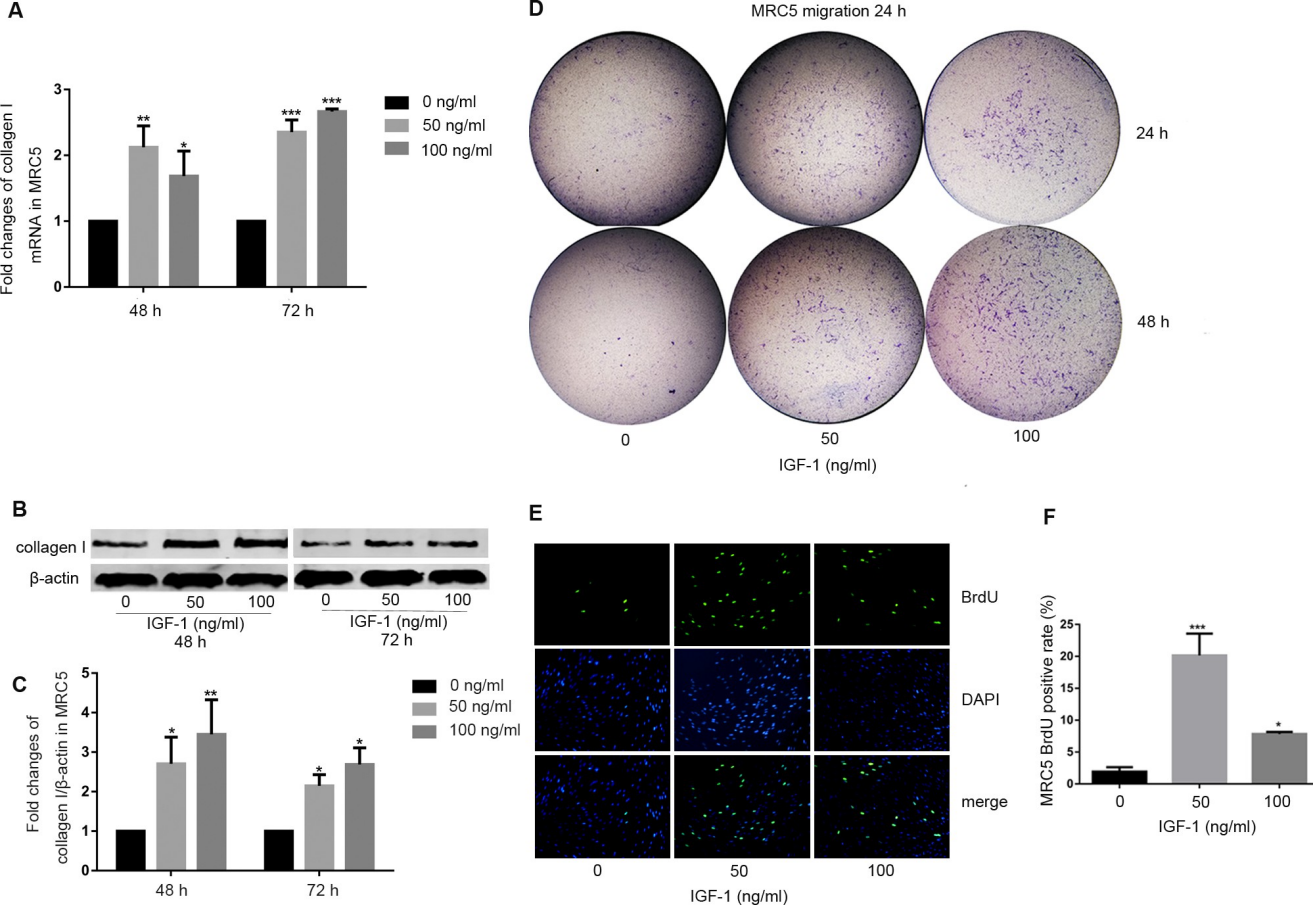
IGF-1 regulated the activation of MRC5 human primary fibroblasts. (A-C) IGF-1 induced collagen I expression of MRC5. MRC5 was treated with or without IGF-1 for 48 or 72 hours. (A) Collagen I mRNA level was measured with real-time PCR. (B and C) Collagen I protein level was determined by western blot analysis and normalized to β-actin. (D) IGF-1 enhanced migration ability of MRC5. MRC5 was treated with or without IGF-1 for 24 or 48 hours. Then MRC5 was harvested, and transwell migration assay was used to assess the 24 hours migration ability of MRC5. (E and F) IGF-1 enhanced proliferation ability of MRC5. (E) MRC5 was treated with IGF-1 for 24 hours, the proliferation of MRC5 was determined by immunofluorescent staining with anti-BrdU (green), and nuclei were stained with DAPI (blue). Original magnification, 200×(E). (F) Quantification of proliferating cells, the chart represents the percentage of BrdU positive cells among the total MRC5. All experiments were performed in triplicate. *P<0.05. **P<0.01, ***P<0.001.

**Fig 7:**
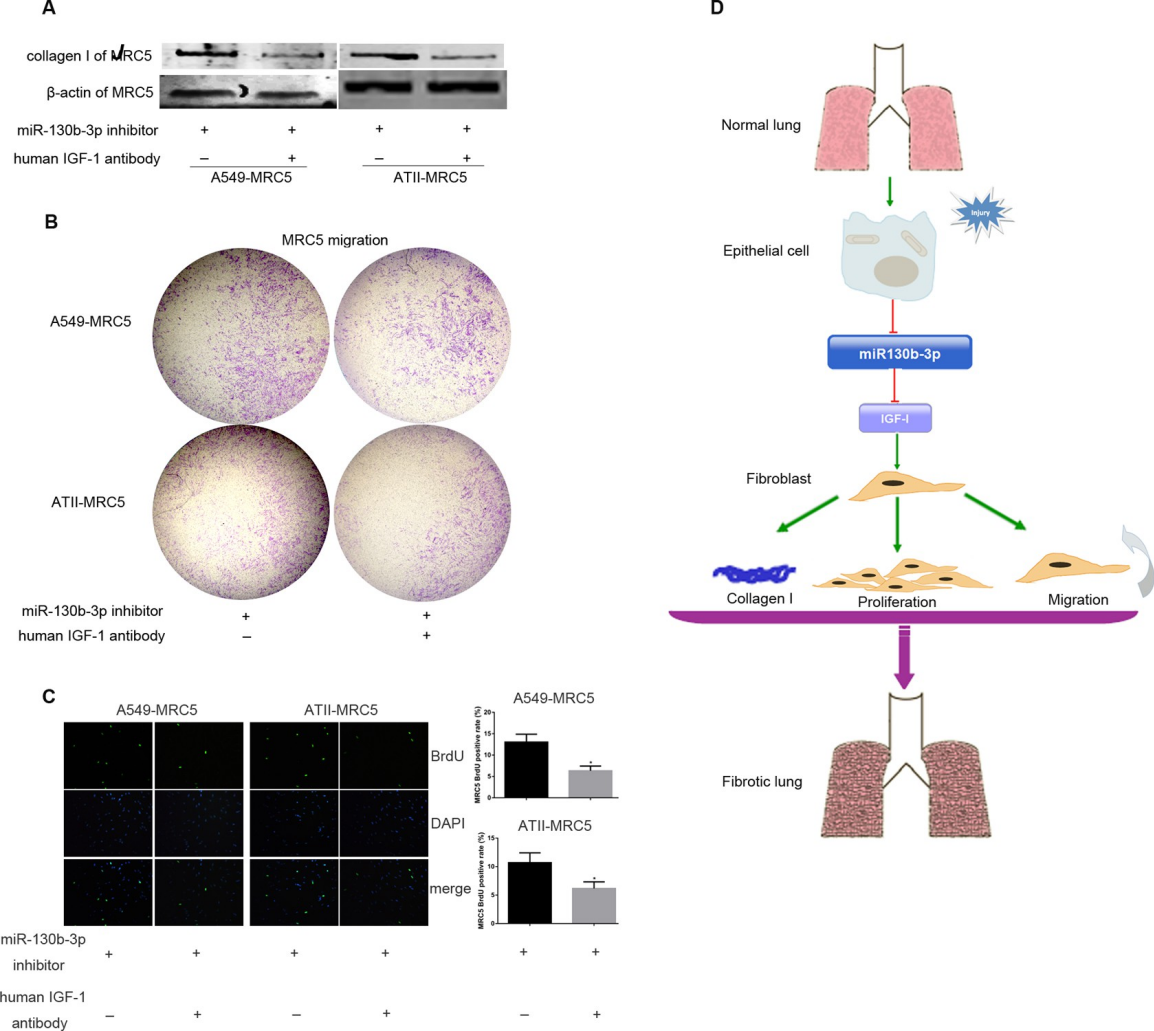
Human IGF-1 antibody prevented miR-130b-3p inhibitor-induced profibrotic effects on fibroblasts. A549 or ATII was transfected with 50 nM miR-130b-3p inhibitor, 24 hours after transfection, A549 or ATII was harvested and co-cultured with starved MRC5 with or without human IGF-1 antibody using 0.4 μm co-culture system (A and C) or 8.0 μm co-culture system (B). After 48 hours, the collagen I protein level (A), migration (B), and proliferation (C) of MRC5 was determined. Original magnification, 200×(C). (D) Persistent injury of alveolar epithelial cells caused decreased expression of miR-130b-3p, which in turn failed to depress the expression of IGF-1. IGF-1, acting as a paracrine activator, regulated the activation of fibroblast though miR-130b-3p dependent mechanisms. Altogether, the downregulation of miR-130b-3p in lung may contribute to the development of lung fibrosis. *P<0.05.

## Supporting information

S2 File Blots in Figs 4, 5 and 7. (A) The blots in Fig 4B. (B) The blots in Fig 5D. (C) The blots in Fig 7A.
(TIF)

S3 File Original western blot images provided for Fig 4B. (PDF)

S4 File Original western blot images provided for Fig 5D. (PDF)

S5 File Original western blot images provided for Fig 7A. (PDF)

S6 File Underlying data for Figs 1B, 2C, 2D, 3A, 3B, 4F, 6D, 6F and 7C. Normalized data for Figs 4A and 4C, 5E and 5F.
(ZIP)

S2 Table Summary data underlying the graphs in Fig 2C and 2D. (DOC)

S3 Table Summary data underlying the graphs in Figs 3A and 3B. (DOC)

S4 Table Summary data underlying the graphs in Fig 4A. (DOC)

S5 Table Summary data underlying the graphs in Fig 4C. (DOC)

S6 Table Summary data underlying the graphs in Fig 4F. (DOC)

S7 Table Summary data underlying the graphs in Fig 5B and 5C. Updated S8 Table from [1].
(DOC)

S8 Table Summary data underlying the graphs in Fig 5E and 5F. Updated S7 Table from [1].
(DOC)

S9 Table Summary data underlying the graphs in Fig 6D and 6F. (DOC)

S10 Table Summary data underlying the graphs in Fig 7C. (DOC)
