# Supplementary figures and images for: miR-130b-3p Modulates Epithelial-Mesenchymal Crosstalk in Lung Fibrosis by Targeting IGF-1
Source: PLoS One. 2016 Mar 8;11(3):e0150418. doi: 10.1371/journal.pone.0150418 (PMC4783101; doi:10.1371/journal.pone.0150418)

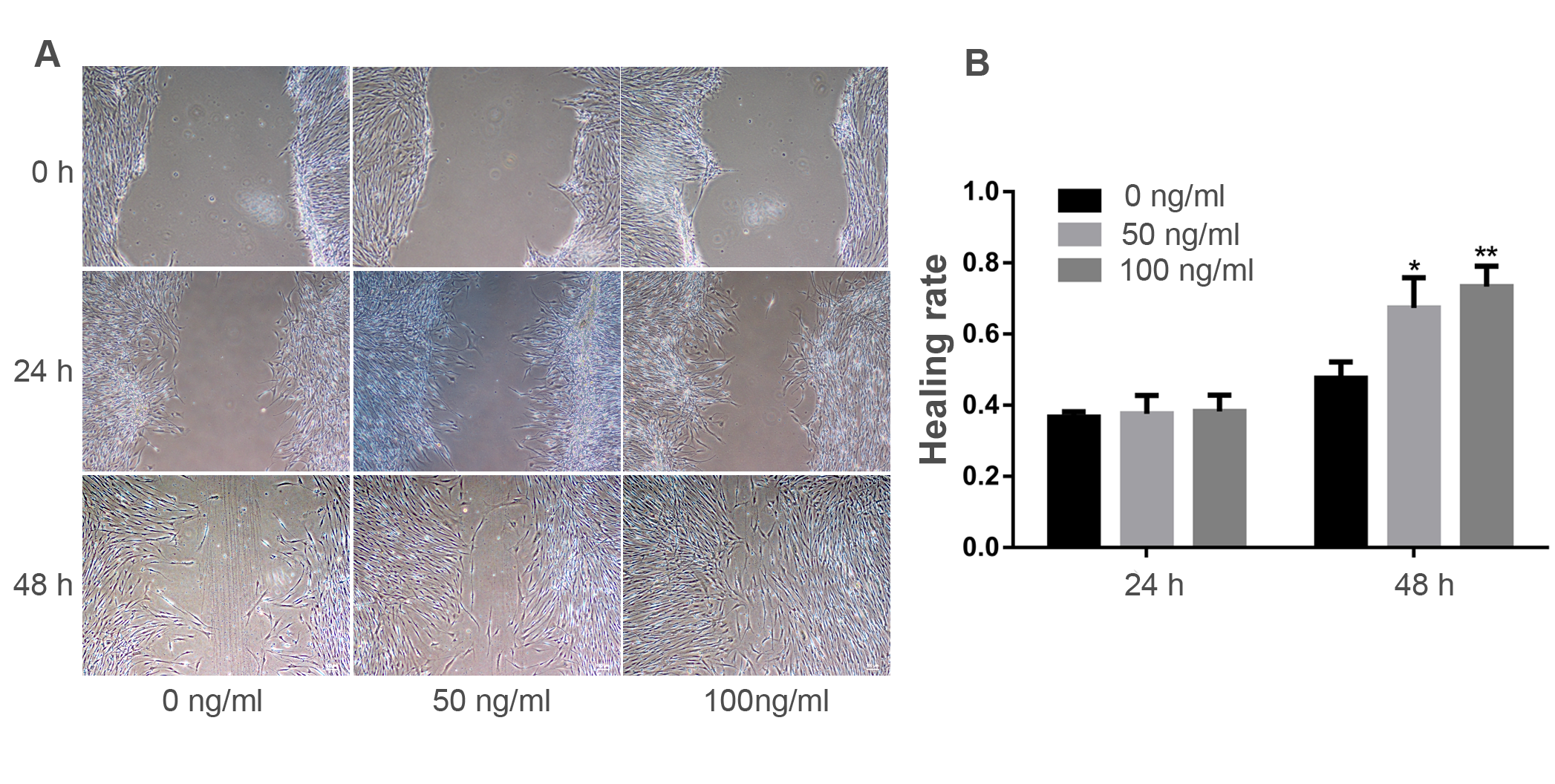

Supplement: S1 Fig — Serum starved MRC5 were incubated for 24 hours and 48 hours in the absence or presence of IGF-1 in alpha-MEM containing 0.1% FBS. (A) Wound healing assay was used to indirectly measure the migratory capacity of MRC5 in six-well plates. Time points 0, 24, 48 hours were observed at 40× magnification. (B) Summary of wound assay results. The average healing distances were measured at three different points per group with the changes of time. *P<0.05, *P<0.01. (TIF) [file pone.0150418.s001.tif]

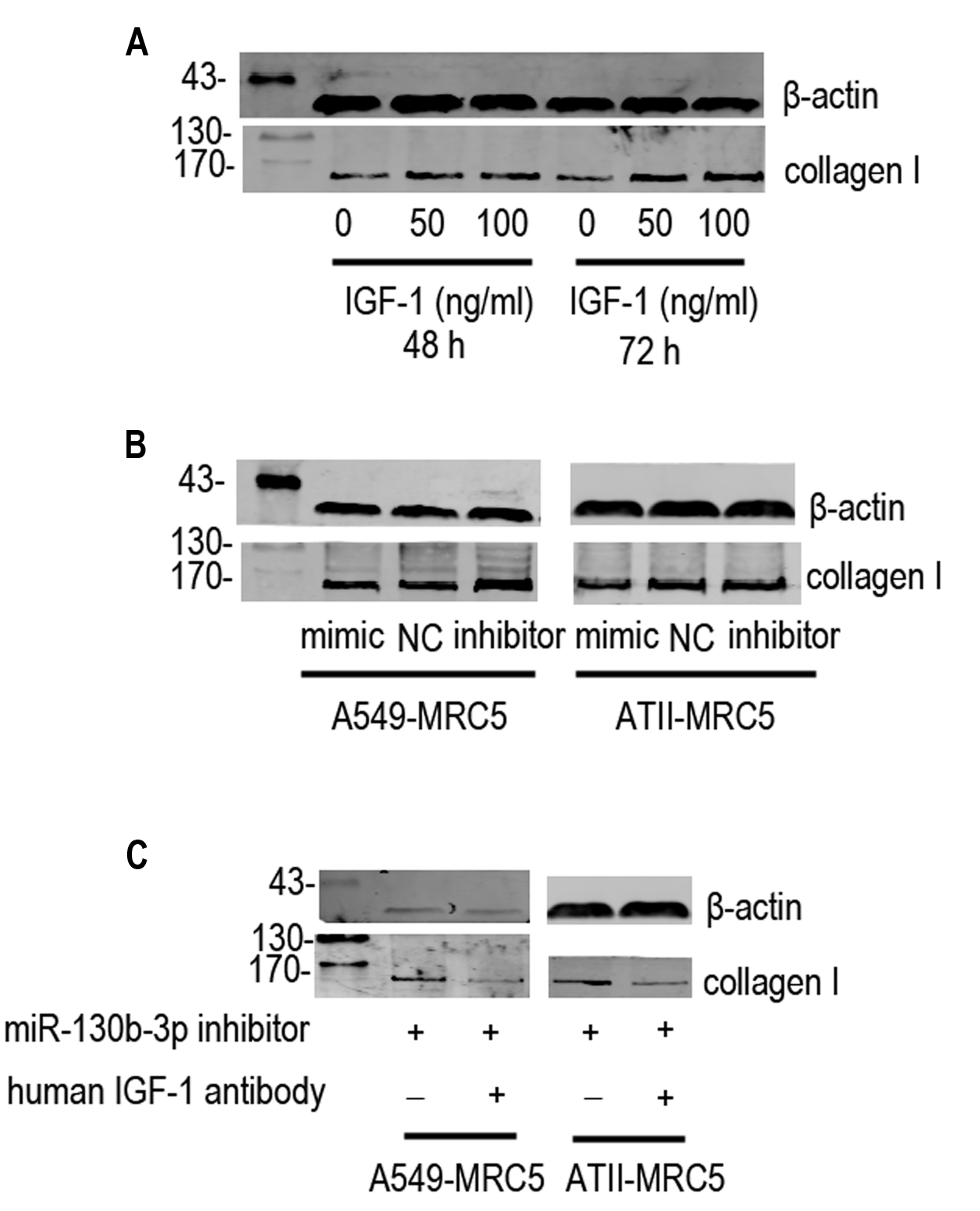

Supplement: S2 File — (A) The original blots in Fig 4B. (B) The original blots in Fig 5D. (C) The original blots in Fig 7A. (TIF) [file pone.0150418.s003.tif]
