# Supplementary material for: miR-130b-3p Modulates Epithelial-Mesenchymal Crosstalk in Lung Fibrosis by Targeting IGF-1
Source: PLoS One. 2016 Mar 8;11(3):e0150418. doi: 10.1371/journal.pone.0150418 (PMC4783101; doi:10.1371/journal.pone.0150418)
Supplement: S1 Table — (DOC) [file pone.0150418.s004.doc]

**S1.Table. Primer sequences (5’-3’) on the products of the PCR and RT-qPCR assay.**

| Gene | Primer sequences |
| --- | --- |
| IGF-1-WT1 | F: 5’-GCGGCTCGAGAAGAGTGACATGCCACCGCA-3’ |
|  | R: 5’-AATGCGGCCGCCAGCAGCCAAGATTCAGAGAG-3’ |
| IGF-1-WT2 | F: 5’-GGCGGCTCGAGGTTTATGAATTGTTTCCTTA -3’ |
|  | R:5’-AATGCGGCCGCACATTAACTCATCATTTGAA -3’ |
| IGF-1-MUT1 | F: 5’-GCGGCTCGAGAAGAGTGACATGCCACCGCA-3’ |
|  | R:5’-AATGCGGCCGCCAGCAGCCAAGATTCAGAGGAATT |
|  | TTCACGTTCTGGATCTATACA-3’ |
| IGF-1-MUT2 | F: 5’-GGCGGCTCGAGGTTTATGAATTGTTTCCTTATAACG |
|  | TGATCTTTCTACACAACTCGG -3’ |
|  | R:5’-AATGCGGCCGCACATTAACTCATCATTTGAA -3’ |
| Collagen I | F: 5’-GGGCAAGACAGTGATTGAATA-3’ |
|  | R: 5’-ACGTCGAAGCCGAATTCCT- 3’ |
| β-actin | F: 5’-TGCTATCCAGGCTGTGCTAT-3’ |
|  | R: 5’-AGTCCATCACGATGCCAGT- 3’ |
| miR-130b-3p | RiboBio |
| U6 | RiboBio |
