# Supplementary material for: miR-130b-3p Modulates Epithelial-Mesenchymal Crosstalk in Lung Fibrosis by Targeting IGF-1
Source: PLoS One. 2016 Mar 8;11(3):e0150418. doi: 10.1371/journal.pone.0150418 (PMC4783101; doi:10.1371/journal.pone.0150418)
Supplement: S2 Table — (DOC) [file pone.0150418.s005.doc]

S2 Table. The data points underlying the graphs in Figs 2C and 2D (means ± SEM, n=3).

| Group | WT-1 | MUT-1 | WT-2 | MUT-2 |
| --- | --- | --- | --- | --- |
| NC | 1.00±0.06 | 1.00±0.05 | 1.00±0.06 | 1.00±0.04 |
| miR-130b-3p | 0.57±0.01^a^ | 0.96±0.04 | 0.66±0.01^a^ | 0.85±0.01 |

^a^*P*<0.01 *vs* NC
