# Supplementary material for: miR-130b-3p Modulates Epithelial-Mesenchymal Crosstalk in Lung Fibrosis by Targeting IGF-1
Source: PLoS One. 2016 Mar 8;11(3):e0150418. doi: 10.1371/journal.pone.0150418 (PMC4783101; doi:10.1371/journal.pone.0150418)
Supplement: S4 Table — (DOC) [file pone.0150418.s007.doc]

S4 Table. The data points underlying the graph in Fig 4A (means ± SEM, n=3).

| Group | 0 ng/ml | 50 ng/ml | 100 ng/ml |
| --- | --- | --- | --- |
| 48 h | 1.00±0.00 | 2.12±0.19^b^ | 1.60±0.22^a^ |
| 72 h | 1.00±0.00 | 2.35±0.11^c^ | 2.67±0.02^c^ |

^a^*P*<0.05 *vs* 0 ng/ml, ^b^*P*<0.01 *vs* 0 ng/ml, ^c^*P*<0.001 *vs* 0 ng/ml
