# Supplementary material for: miR-130b-3p Modulates Epithelial-Mesenchymal Crosstalk in Lung Fibrosis by Targeting IGF-1
Source: PLoS One. 2016 Mar 8;11(3):e0150418. doi: 10.1371/journal.pone.0150418 (PMC4783101; doi:10.1371/journal.pone.0150418)
Supplement: S5 Table — (DOC) [file pone.0150418.s008.doc]

S5 Table. The data points underlying the graph in Fig 4C (means ± SEM, n=3).

| Group | 0 ng/ml | 50 ng/ml | 100 ng/ml |
| --- | --- | --- | --- |
| 48 h | 1.00±0.00 | 2.71±0.34^a^ | 3.45±0.50^b^ |
| 72 h | 1.00±0.00 | 2.15±0.16^a^ | 2.69±0.24^a^ |

^a^*P*<0.05 *vs* 0 ng/ml, ^b^*P*<0.01 *vs* 0 ng/ml
