# Supplementary material for: miR-130b-3p Modulates Epithelial-Mesenchymal Crosstalk in Lung Fibrosis by Targeting IGF-1
Source: PLoS One. 2016 Mar 8;11(3):e0150418. doi: 10.1371/journal.pone.0150418 (PMC4783101; doi:10.1371/journal.pone.0150418)
Supplement: S6 Table — (DOC) [file pone.0150418.s009.doc]

S6 Table. The data points underlying the graph in Fig 4F (means ± SEM, n=3).

| 0 ng/ml | 50 ng/ml | 100 ng/ml |
| --- | --- | --- |
| 1.92±0.41 | 19.97±2.14^b^ | 7.84±0.19^a^ |

^a^*P*<0.05 *vs* 0 ng/ml, ^b^*P*<0.001 *vs* 0 ng/ml
