# Supplementary material for: miR-130b-3p Modulates Epithelial-Mesenchymal Crosstalk in Lung Fibrosis by Targeting IGF-1
Source: PLoS One. 2016 Mar 8;11(3):e0150418. doi: 10.1371/journal.pone.0150418 (PMC4783101; doi:10.1371/journal.pone.0150418)
Supplement: S7 Table — (DOC) [file pone.0150418.s010.doc]

S7 Table. The data points underlying the graphs in Figs 5B and 5C (means ± SEM, n=3).

| Group | A549 | ATII |
| --- | --- | --- |
| miR-130b-3p mimic | 1.00±0.00 | 1.00±0.00 |
| miR-130b-3p NC | 1.32±0.04^a^ | 1.37±0.65^a^ |
| miR-130b-3p inhibitor | 2.90±0.06^c^ | 1.80±0.58^b^ |

^a^*P*<0.05 *vs* mimic*,* ^b^*P*<0.05 *vs* NC, ^c^*P*<0.01 *vs* NC
