# Supplementary material for: miR-130b-3p Modulates Epithelial-Mesenchymal Crosstalk in Lung Fibrosis by Targeting IGF-1
Source: PLoS One. 2016 Mar 8;11(3):e0150418. doi: 10.1371/journal.pone.0150418 (PMC4783101; doi:10.1371/journal.pone.0150418)
Supplement: S9 Table — (DOC) [file pone.0150418.s012.doc]

S9 Table. The data points underlying the graphs in Figs 6D and 6F (means ± SEM, n=3).

| Group | A549 | ATII |
| --- | --- | --- |
| miR-130b-3p mimic | 15.46±0.29 | 4.72±0.12 |
| miR-130b-3p NC | 25.95±0.13^a^ | 10.87±1.49^a^ |
| miR-130b-3p inhibitor | 41.08±5.12^b^ | 17.06±1.20^b^ |

^a^*P*<0.05 *vs* mimic*,* ^b^*P*<0.05 *vs* NC
