# Supplementary material for: miR-130b-3p Modulates Epithelial-Mesenchymal Crosstalk in Lung Fibrosis by Targeting IGF-1
Source: PLoS One. 2016 Mar 8;11(3):e0150418. doi: 10.1371/journal.pone.0150418 (PMC4783101; doi:10.1371/journal.pone.0150418)
Supplement: S10 Table — (DOC) [file pone.0150418.s013.doc]

S10 Table. The data points underlying the graphs in Fig 7C (means ± SEM, n=3).

| Group | A549 | ATII |
| --- | --- | --- |
| miR-130b-3p inhibitor | 13.03±1.07 | 10.71±0.99 |
| Human IGF-1 antibody | 6.35±0.63^a^ | 6.51±0.82^a^ |

^a^*P*<0.05 *vs* miR-130b-3p inhibitor
