# Supplementary figures and images for: Expression of Concern: miR-130b-3p Modulates Epithelial-Mesenchymal Crosstalk in Lung Fibrosis by Targeting IGF-1
Source: PLoS One. 2022 Feb 3;17(2):e0263701. doi: 10.1371/journal.pone.0263701 (PMC8812954; doi:10.1371/journal.pone.0263701)

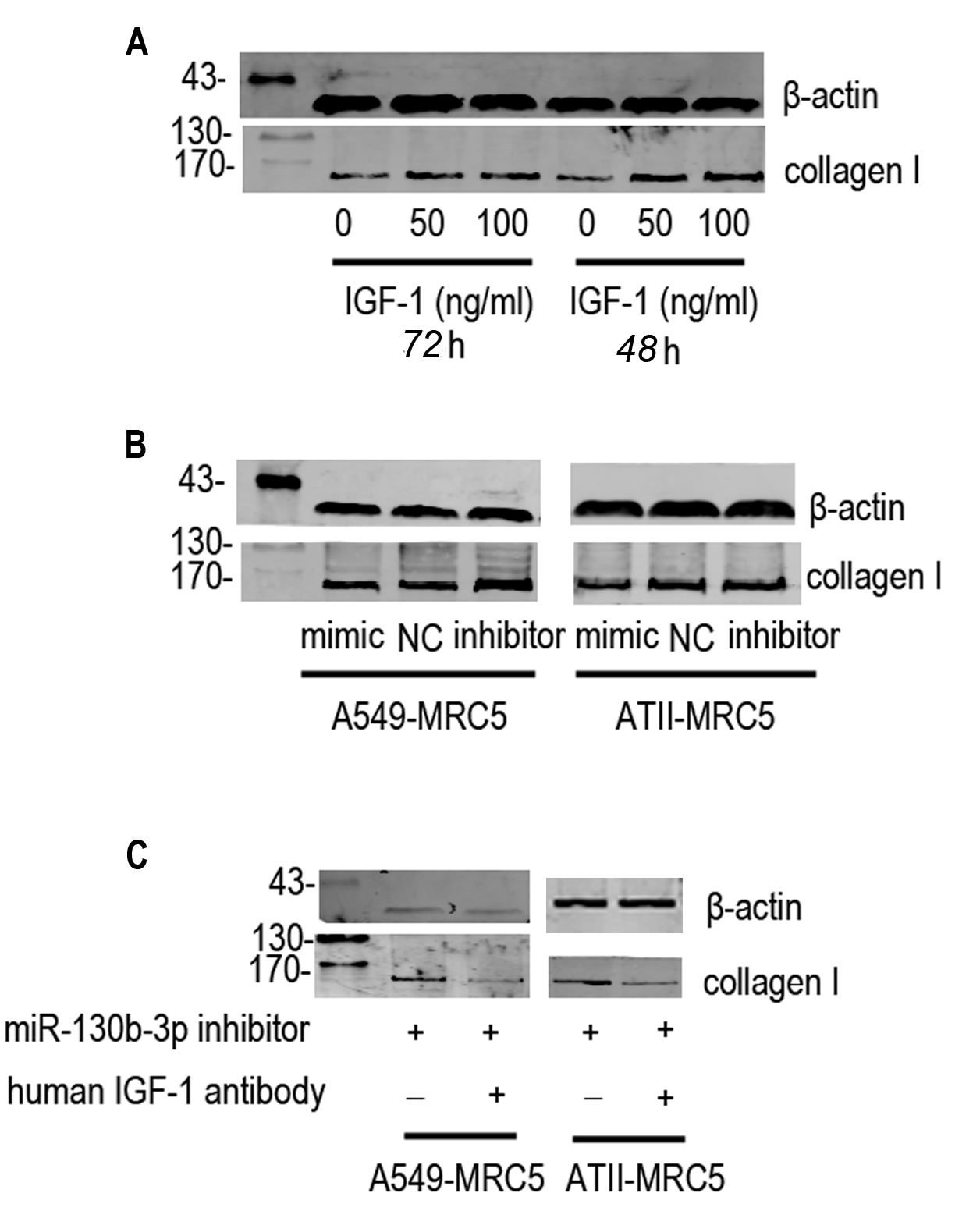

Supplement: S2 File — (A) The blots in Fig 4B. (B) The blots in Fig 5D. (C) The blots in Fig 7A. (TIF) [file pone.0263701.s001.tif]

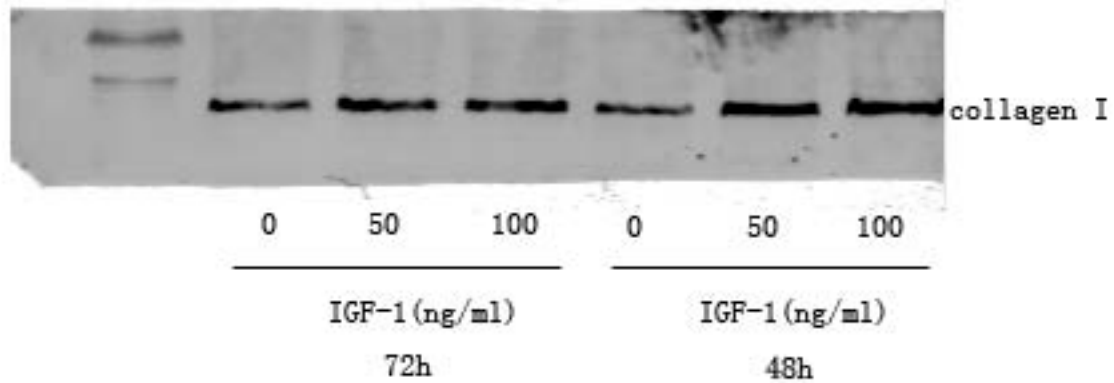

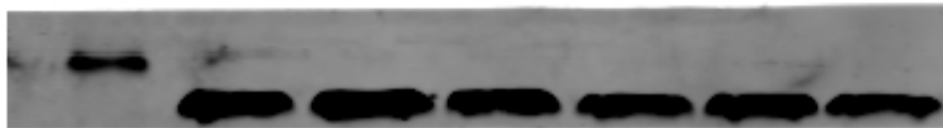

0

50

100

0

50

100

IGF-1 (ng/ml)

IGF-1 (ng/ml)

72h

48h

Supplement: S3 File — (PDF) [file pone.0263701.s002.pdf]
