# Supplementary material for: Expression of Concern: miR-130b-3p Modulates Epithelial-Mesenchymal Crosstalk in Lung Fibrosis by Targeting IGF-1
Source: PLoS One. 2022 Feb 3;17(2):e0263701. doi: 10.1371/journal.pone.0263701 (PMC8812954; doi:10.1371/journal.pone.0263701)
Supplement: S4 File — (PDF) [file pone.0263701.s003.pdf]

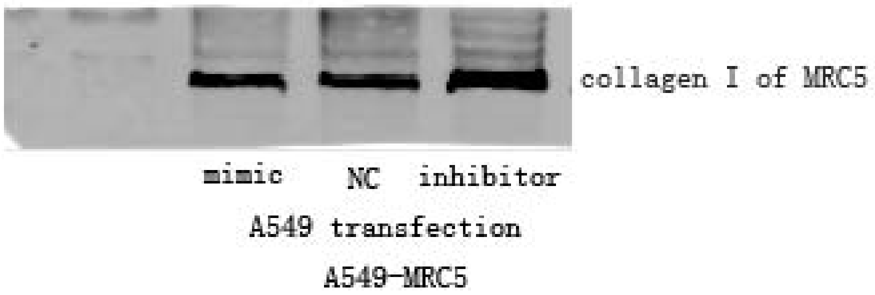
collagen I of MRC5
mimic
NC
inhibitor
A549 transfection
A549-MRC5

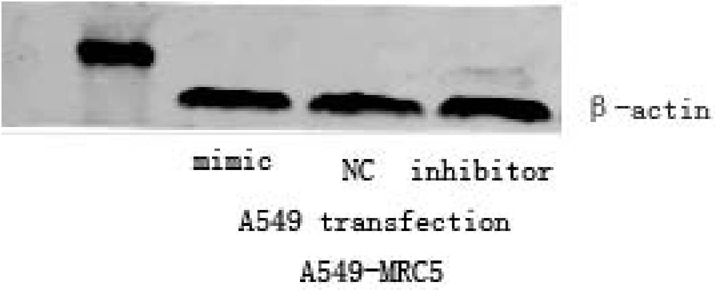
β-actin
mimic
NC
inhibitor
A549 transfection
A549-MRC5

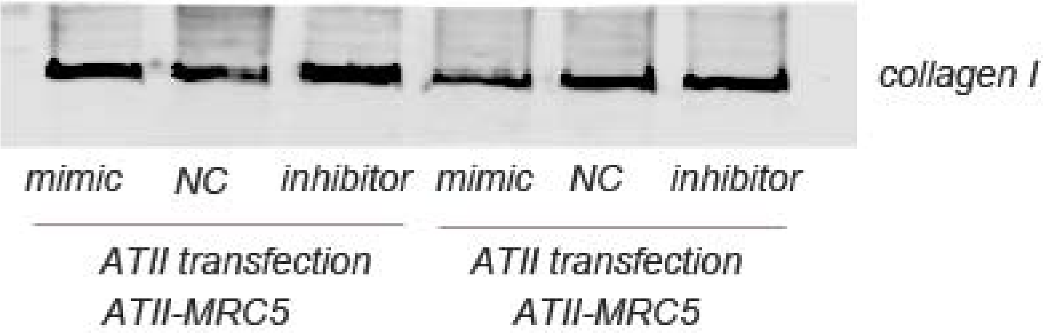
collagen I
mimic
NC
inhibitor
mimic
NC
inhibitor
ATII transfection
ATII-MRC5
ATII transfection
ATII-MRC5

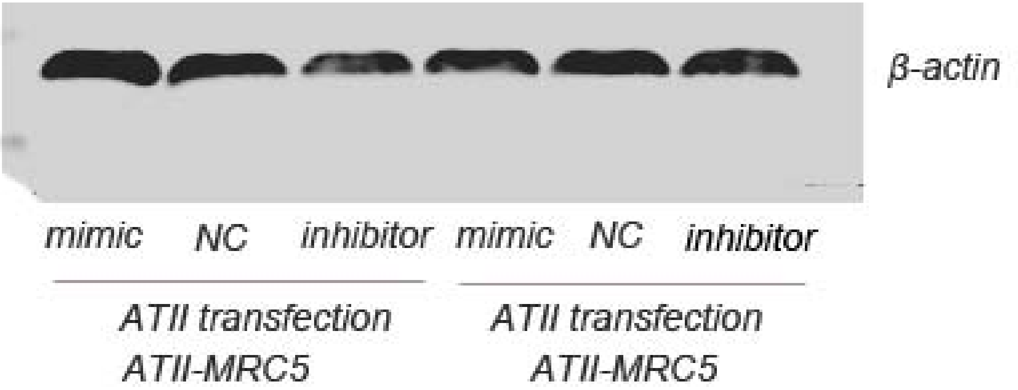
β-actin
mimic
NC
inhibitor
mimic
NC
inhibitor
ATII transfection
ATII-MRC5
ATII transfection
ATII-MRC5
