# Supplementary material for: Expression of Concern: miR-130b-3p Modulates Epithelial-Mesenchymal Crosstalk in Lung Fibrosis by Targeting IGF-1
Source: PLoS One. 2022 Feb 3;17(2):e0263701. doi: 10.1371/journal.pone.0263701 (PMC8812954; doi:10.1371/journal.pone.0263701)
Supplement: S5 File — (PDF) [file pone.0263701.s004.pdf]

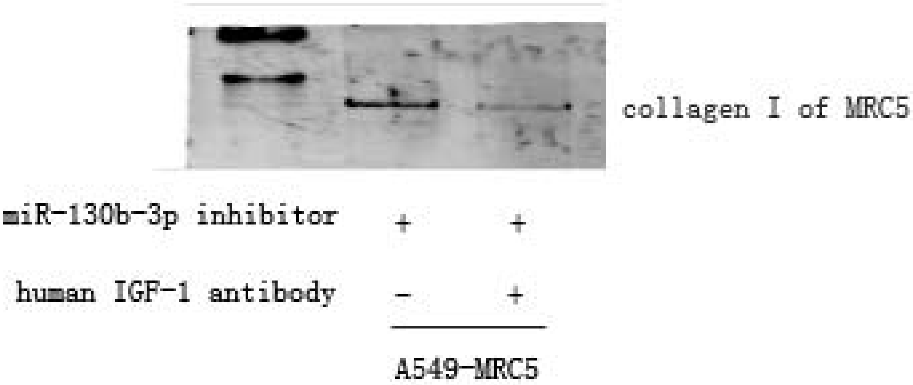
collagen I of MRC5
miR-130b-3p inhibitor + +
human IGF-1 antibody − +
A549-MRC5

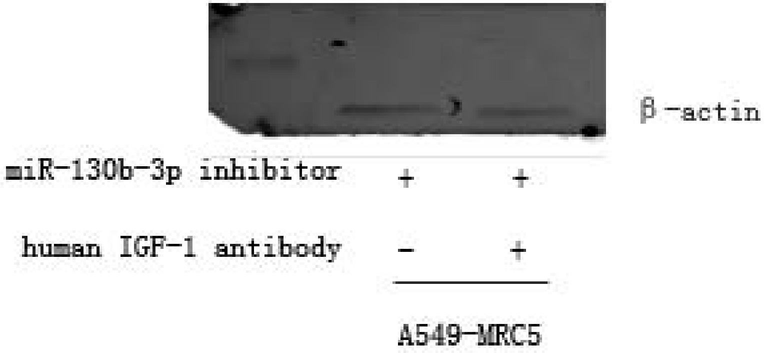
β-actin
miR-130b-3p inhibitor + +
human IGF-1 antibody - +
A549-MRC5

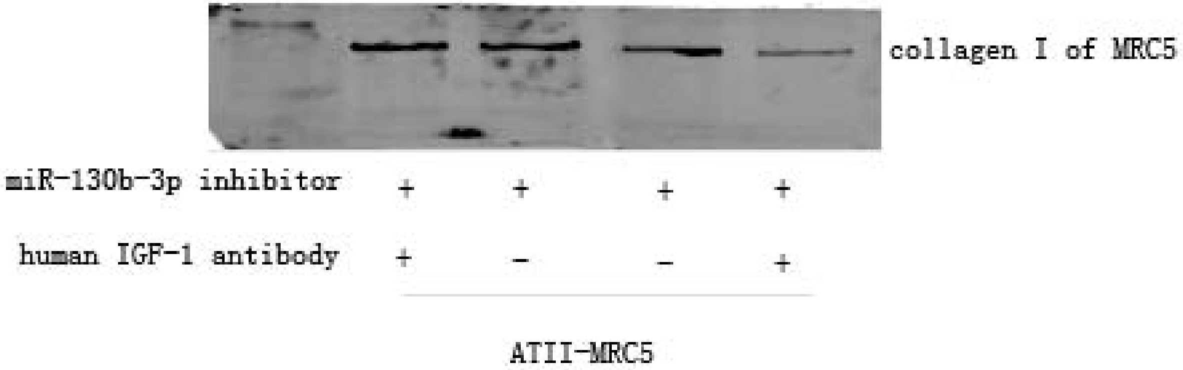

collagen I of MRC5
miR-130b-3p inhibitor + + + +
human IGF-1 antibody + − − +
ATII-MRC5

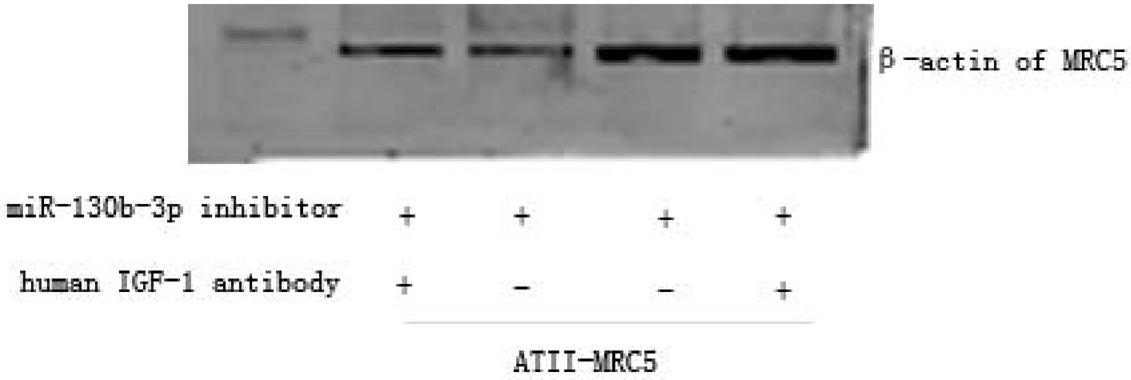
β-actin of MRC5
miR-130b-3p inhibitor + + + +
human IGF-1 antibody + − − +
ATII-MRC5
