# Supplementary material for: Expression of Concern: miR-130b-3p Modulates Epithelial-Mesenchymal Crosstalk in Lung Fibrosis by Targeting IGF-1
Source: PLoS One. 2022 Feb 3;17(2):e0263701. doi: 10.1371/journal.pone.0263701 (PMC8812954; doi:10.1371/journal.pone.0263701)
Supplement: S2 Table — (DOC) [file pone.0263701.s006.doc]

S2 Table. Summary data underlying the graphs in Figs 2C and 2D (means ± SEM, n=3).

| Group | WT-1 | MUT-1 | WT-2 | MUT-2 |
| --- | --- | --- | --- | --- |
| NC | 1.00±0.04 | 1.00±0.03 | 1.00±0.04 | 1.00±0.03 |
| miR-130b-3p | 0.57±0.01^a^ | 0.96±0.02 | 0.66±0.01^a^ | 0.85±0.01 |

^a^*P*<0.01 *vs* NC
