# Supplementary material for: Expression of Concern: miR-130b-3p Modulates Epithelial-Mesenchymal Crosstalk in Lung Fibrosis by Targeting IGF-1
Source: PLoS One. 2022 Feb 3;17(2):e0263701. doi: 10.1371/journal.pone.0263701 (PMC8812954; doi:10.1371/journal.pone.0263701)
Supplement: S3 Table — (DOC) [file pone.0263701.s007.doc]

S3 Table. Summary data underlying the graphs in Figs 3A and 3B (means ± SEM, n=3).

| Group | A549 | ATII |
| --- | --- | --- |
| miR-130b-3p mimic | 0.25±0.00 | 0.09±0.00 |
| miR-130b-3p NC | 0.31±0.01^a^ | 0.13±0.01^a^ |
| miR-130b-3p inhibitor | 0.46±0.01^b^ | 0.23±0.01^b^ |

^a^*P*<0.01 *vs* mimic*,* ^b^*P*<0.01 *vs* NC
