# Supplementary material for: Expression of Concern: miR-130b-3p Modulates Epithelial-Mesenchymal Crosstalk in Lung Fibrosis by Targeting IGF-1
Source: PLoS One. 2022 Feb 3;17(2):e0263701. doi: 10.1371/journal.pone.0263701 (PMC8812954; doi:10.1371/journal.pone.0263701)
Supplement: S4 Table — (DOC) [file pone.0263701.s008.doc]

S4 Table. Summary data points underlying the graph in Fig 4A (means ± SEM, n=3).

| Group | 0 ng/ml | 50 ng/ml | 100 ng/ml |
| --- | --- | --- | --- |
| 48 h | 1.00±0.00 | 2.12±0.19^b^ | 1.69±0.22^a^ |
| 72 h | 1.00±0.00 | 2.35±0.11^c^ | 2.67±0.02^c^ |

^a^*P*<0.05 *vs* 0 ng/ml, ^b^*P*<0.01 *vs* 0 ng/ml, ^c^*P*<0.001 *vs* 0 ng/ml
