# Supplementary material for: Expression of Concern: miR-130b-3p Modulates Epithelial-Mesenchymal Crosstalk in Lung Fibrosis by Targeting IGF-1
Source: PLoS One. 2022 Feb 3;17(2):e0263701. doi: 10.1371/journal.pone.0263701 (PMC8812954; doi:10.1371/journal.pone.0263701)
Supplement: S6 Table — (DOC) [file pone.0263701.s010.doc]

S6 Table. Summary data underlying the graph in Fig 4F (means ± SEM, n=3).

| 0 ng/ml | 50 ng/ml | 100 ng/ml |
| --- | --- | --- |
| 1.92±0.41 | 20.17±1.97^b^ | 7.84±0.19^a^ |

^a^*P*<0.05 *vs* 0 ng/ml, ^b^*P*<0.001 *vs* 0 ng/ml
