# Supplementary material for: Expression of Concern: miR-130b-3p Modulates Epithelial-Mesenchymal Crosstalk in Lung Fibrosis by Targeting IGF-1
Source: PLoS One. 2022 Feb 3;17(2):e0263701. doi: 10.1371/journal.pone.0263701 (PMC8812954; doi:10.1371/journal.pone.0263701)
Supplement: S8 Table — Updated S7 Table from [1]. (DOC) [file pone.0263701.s012.doc]

S8 Table. Summary data underlying the graphs in Figs 5E and 5F (means ± SEM, n=3).

| Group | A549 | ATII |
| --- | --- | --- |
| miR-130b-3p mimic | 1.00±0.00 | 1.00±0.00 |
| miR-130b-3p NC | 1.32±0.04^a^ | 1.37±0.07^a^ |
| miR-130b-3p inhibitor | 2.90±0.06^c^ | 1.80±0.06^b^ |

^a^*P*<0.05 *vs* mimic*,* ^b^*P*<0.05 *vs* NC, ^c^*P*<0.01 *vs* NC
