# Supplementary material for: Expression of Concern: miR-130b-3p Modulates Epithelial-Mesenchymal Crosstalk in Lung Fibrosis by Targeting IGF-1
Source: PLoS One. 2022 Feb 3;17(2):e0263701. doi: 10.1371/journal.pone.0263701 (PMC8812954; doi:10.1371/journal.pone.0263701)
Supplement: S9 Table — (DOC) [file pone.0263701.s013.doc]

S9 Table. Summary data underlying the graphs in Figs 6D and 6F (means ± SEM, n=3).

| Group | A549 | ATII |
| --- | --- | --- |
| miR-130b-3p mimic | 15.46±0.29 | 4.72±0.12 |
| miR-130b-3p NC | 25.95±0.13^a^ | 10.87±1.49^a^ |
| miR-130b-3p inhibitor | 41.08±5.12^b^ | 17.06±1.20^b^ |

^a^*P*<0.05 *vs* mimic*,* ^b^*P*<0.05 *vs* NC
