# Supplementary material for: Expression of Concern: miR-130b-3p Modulates Epithelial-Mesenchymal Crosstalk in Lung Fibrosis by Targeting IGF-1
Source: PLoS One. 2022 Feb 3;17(2):e0263701. doi: 10.1371/journal.pone.0263701 (PMC8812954; doi:10.1371/journal.pone.0263701)
Supplement: S10 Table — (DOC) [file pone.0263701.s014.doc]

S10 Table. Summary data underlying the graphs in Fig 7C (means ± SEM, n=3).

| Group | A549 | ATII |
| --- | --- | --- |
| miR-130b-3p inhibitor | 13.03±1.07 | 10.71±0.99 |
| Human IGF-1 antibody | 6.35±0.63^a^ | 6.17±0.67^a^ |

^a^*P*<0.05 *vs* miR-130b-3p inhibitor
